# Physical exercise increases binding of POMC to blood extracellular vesicles

**DOI:** 10.1073/pnas.2525044122

**Published:** 2025-12-16

**Authors:** Mark F. Santos, Jacqueline Randa, Derek Tai, Giulio Vistoli, Nofar Avihen Schahaf, Serena Vittorio, Geily Fuentes, Rita Lauro, Sheila Mosallaei, Jana Karbanová, Alexandra M. K. Yokomizo, Denis Corbeil, Cheryl E. Hightower, Aurelio Lorico

**Affiliations:** ^a^Department of Basic Sciences, College of Osteopathic Medicine, Touro University Nevada, Henderson, NV 89014; ^b^Department of Physical Therapy, College of Health and Human Health Sciences, Touro University Nevada, Henderson, NV 89014; ^c^Department of Pharmaceutical Sciences, University of Milan, Milan 20122, Italy; ^d^Department of Biomedical Sciences, Dentistry and Morphological and Functional Images, University of Messina, Messina 98122, Italy; ^e^Tissue Engineering, Biotechnology Center and Center for Molecular and Cellular Bioengineering, Technische Universität Dresden, Dresden 01307, Germany; ^f^Tissue Engineering Laboratories, Medizinische Fakultät der Technischen Universität Dresden, Dresden 01307, Germany; ^g^Imgen, Research Group, Las Vegas, NV 89117

**Keywords:** exosomes, pro-opiomelanocortin, endorphin, melanocortin, hormone receptor

## Abstract

Physical exercise affects multiple physiological processes, including those regulated by hormones, such as energy balance, stress responses, and pain perception. The prohormone precursor proopiomelanocortin (POMC) gives rise to β-endorphin, adrenocorticotropic hormone, β-lipotropin, and the melanocyte-stimulating hormones (MSH), which act through melanocortin (MC1-5) and opioid receptors to influence these key physiological responses. Here, we identified a mechanism by which full-length POMC interacts with small extracellular vesicles (sEVs) in the circulation. In healthy adults, the fraction of plasma POMC associated with sEVs increased fourfold following intense exercise, despite unchanged POMC and sEV concentrations. POMC-positive sEVs contained leukocyte or megakaryocyte/platelet markers, suggesting hematopoietic origin. A substantial subset of circulating sEVs expressed MC1–MC5 and μ-opioid receptors, and coimmunoisolation assays demonstrated binding of unprocessed POMC to MC1 and MC3 receptors. Molecular modeling indicated that POMC undergoes pH-dependent conformational changes that favor MC binding, a finding corroborated by in vitro binding assays under acidic conditions. Functionally, addition of POMC-positive sEVs to B16-F10 melanoma cells induced melanogenesis, consistent with POMC-derived MSH activity. The sEV-associated POMC had higher rates of transport than unbound POMC in assays mimicking the blood–brain barrier. These findings establish that exercise-induced, pH-dependent binding of POMC to circulating sEVs may facilitate hormone precursor transport and potentially influence transfer across the blood–brain barrier. This mechanism represents a separate paradigm for endocrine regulation, providing insight into how exercise modulates sEV interactions with systemic hormone distribution and signaling.

## Significance

Mechanisms underlying interactions between small extracellular vesicles (sEVs) and hormones remain largely unexplored. Here, we identify an interaction between the full-length hormone precursor proopiomelanocortin (POMC) and circulating sEVs, where POMC binds to sEV membranes via G-protein-coupled receptors. Vigorous exercise did not change plasma concentration of POMC or sEVs; however, exercise-induced blood acidification increased POMC–sEV association by fourfold by promoting a conformational change in POMC that favored sEV binding. Physiologically, the interactions may facilitate tissue distribution and transfer of hormones across the blood–brain barrier, relative to unbound, soluble POMC. These findings uncover a stress-inducible mechanism that fine-tunes endocrine signaling under conditions of physiological stress.

Increasing evidence supports a central role for small extracellular vesicles (sEVs) in intercellular communication and their interactions with the endocrine system (1, 2). sEVs are nanosized (30 to 200 nm in diameter) membrane-bound structures present at concentrations of billions per milliliter in all body fluids (3). Cells release sEVs either as exosomes, originating from the endocytic system, or as ectosomes/microvesicles, budding directly from the plasma membrane (3). sEVs transport proteins, lipids, and nucleic acids as intraluminal cargo, but can also display or associate with biomolecules on their surface (2). Mechanisms of sEV-mediated communication are not fully understood, with evidence supporting both transfer of RNAs and proteins carried in the lumen (4) and membrane-mediated interactions with cell surface receptors (5). Here, we investigated potential interactions between circulating sEVs and the prohormone proopiomelanocortin (POMC) by focusing on stressful conditions when hormone-regulated physiological processes are activated.

The prohormone POMC is detectable in the circulation (6), and undergoes posttranslational proteolytic cleavage to release multiple bioactive peptides, including adrenocorticotropic hormone (ACTH), melanocyte-stimulating hormones (α-MSH, β–MSH, and γ-MSH), and β-endorphin, through the regulated pathway (7). Unprocessed POMC is found at higher concentrations in the blood than ACTH (8–10), and is likely released through a constitutive-like pathway (11). Although POMC can initiate a response in some cells, such as melanin production in melanocytes (7), the function of blood-borne POMC beyond its role as prohormone has not been fully characterized. Moreover, interactions between POMC and other circulating components such as sEVs are not well understood.

Previous studies have reported that physical exercise increases circulating levels of POMC-derived hormones (12, 13), and EVs have also been implicated in mediating some of the positive effects of exercise (14). These observations prompted us to investigate whether POMC associates with sEVs in response to the systemic stress induced by vigorous exercise, with the objective of gaining mechanistic insight into potential sEV–POMC interactions. Exercise exerts widespread benefits on human health, many of which are partly mediated by endocrine function (2), yet how exercise modulates hormone dynamics is unclear. POMC is of particular interest relative to exercise, as its derived mature hormones regulate multiple exercise-affected physiological processes, including pain perception, glucocorticoid production, stress responses, blood pressure, energy balance, obesity risk, and immune functions (7, 15).

Here, we report the finding that a fraction of plasma POMC is carried by sEVs in healthy adults, and that this fraction increased by fourfold during intense physical exercise, despite unchanged plasma POMC and sEV concentrations. Full-length POMC association with sEVs was promoted by exercise-induced acidification of the blood, which modified the POMC conformation and enhanced its binding to melanocortin receptors on the sEV surface. Such interactions may alter the activity of POMC-derived peptides and facilitate passage of POMC across membranes, including the blood–brain barrier (BBB). These results reveal a mechanism of interaction between a prohormone and sEVs that increases by exercise.

## Results

### Detection of POMC and POMC-Derived Hormones in Plasma and sEVs before and after Exercise.

**Fig. 1.**
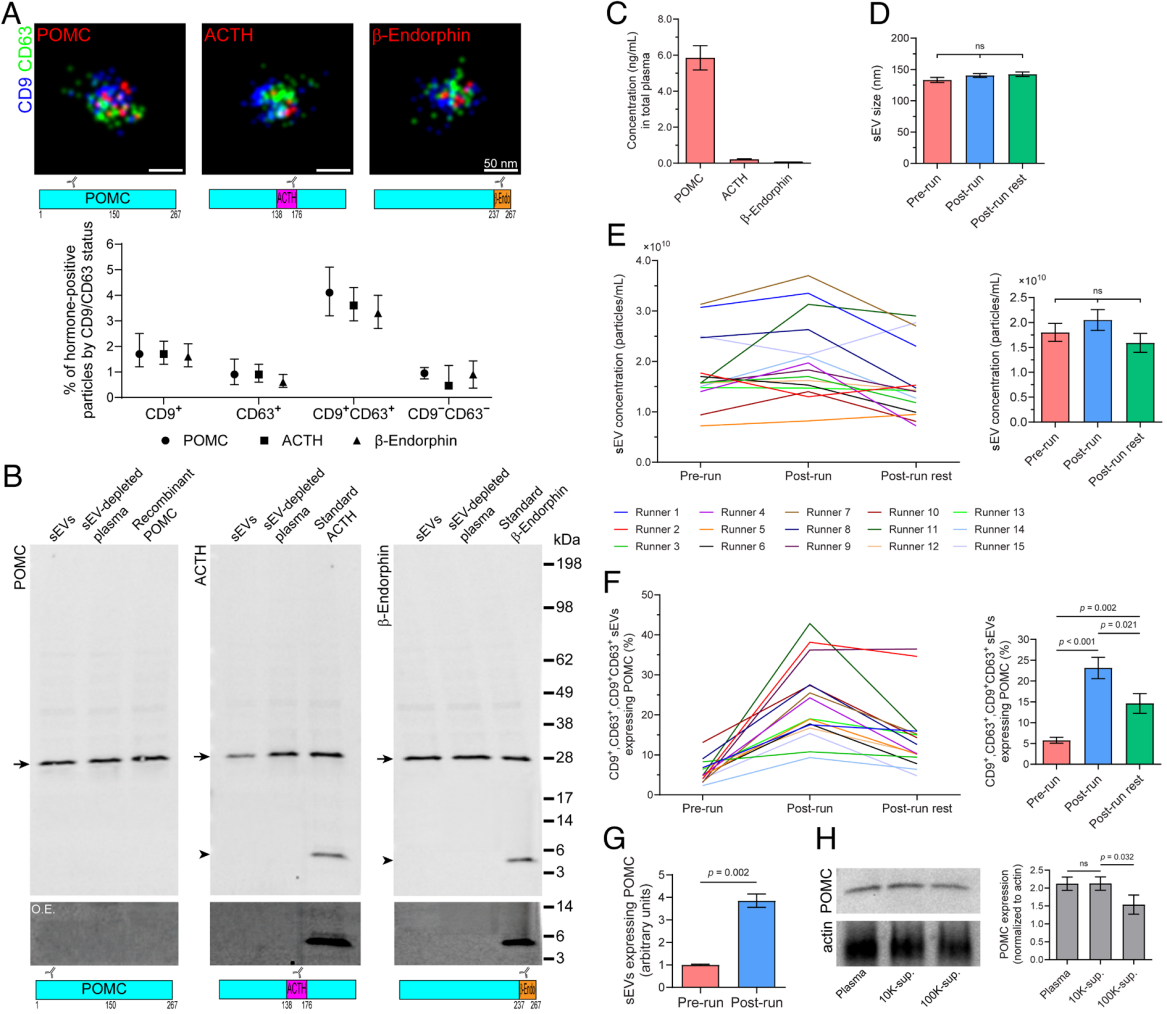
The fraction of POMC associated with plasma sEVs increases after intense physical exercise. (*A*) sEVs isolated from prerun plasma samples were analyzed by d-STORM after triple immunolabeling for CD9 (blue), CD63 (green), and POMC, ACTH, or β-endorphin (red). Representative d-STORM images are shown in the *Upper* panel. The relative position of the epitope of each Ab is displayed in the cartoon of POMC below the images. The percentage of sEVs expressing a given specific hormone within CD9^+^, CD63^+^, CD9^+^CD63^+^, or CD9^−^CD63^−^ populations were quantified (*Lower* panel, n = 3 runners, mean ± 95% CI). (*B*) sEVs isolated from plasma, and fractions corresponding to sEVs and sEV-depleted plasma were analyzed by immunoblotting for POMC (arrow), ACTH or β-endorphin (arrowhead). Recombinant or standard proteins were used as controls. Molecular mass markers (kDa) are indicated. An area of the blot corresponding to 3 to 14 kDa range was overexposed (O.E.). The relative position of the epitope of each Ab is displayed in the cartoon of POMC below the images. (*C*) Plasma concentrations of POMC, ACTH, and β-endorphin in plasma before exercise (prerun), as measured by ELISA (n = 3 runners, mean ± SEM). (*D* and *E*) Size (*D*) and concentration (*E*) of sEVs isolated from the blood of runners across exercise phases (prerun, postrun, postrun rest) were measured using NTA. Line graph (*E*, *Left*) shows individual data for each runner, while bar graphs (*Right*) represent the combined data (n = 15, mean ± SEM) across all participants. No significant differences (*p* > 0.05) were observed between conditions. (*F*) Percent of plasma-derived CD9^+^, CD63^+^, or CD9^+^/CD63^+^ sEVs that were POMC^+^, across exercise phases by d-STORM. Data from individual runners (*Left*) and combined data for all participants (*Right*, n = 15 runners, mean ± SEM) are presented. (*G*) The POMC concentration in the sEVs of prerun and postrun plasma from runners was measured by ELISA (n = 4 runners, mean ± SEM). (*H*) Postrun total plasma, plasma after 10,000 × g centrifugation (10K-sup.), and plasma after 100,000 × g centrifugation (100K-sup.) were analyzed by immunoblotting for POMC and quantified relative to actin. Representative immunoblots are shown, and quantification is presented as mean ± SEM (n = 3). *P*-values are indicated (*Statistical Methods*).

To investigate whether POMC and/or its mature peptide hormones associate with circulating sEVs, we enriched sEVs from plasma of healthy volunteers, and characterized them following MISEV 2023 guidelines (see *Materials and Methods* section: *Isolation and Characterization of sEVs*; *SI Appendix*, Fig. S1) (16, 17). We then used direct stochastic optical reconstruction microscopy (d-STORM) to visualize, at high resolution, the potential association of POMC with individual sEVs (Fig. 1*A*). The sEV surface was immunolabeled with the tetraspanins CD9 and CD63, commonly used sEV markers (18), while POMC, ACTH, and β-endorphin were detected using three distinct antibodies (Abs) (Fig. 1*A*, see cartoon). Between 4% and 8% of sEVs expressing CD9 or CD63 or both were immunoreactive for POMC, ACTH, or β-endorphin (Fig. 1 *A*, *Lower*). A minute fraction of hormone-positive EVs were negative for both sEV markers: 0.95% (POMC, 95% CI: 0.73 to 1.17); 0.46% (ACTH, 95% CI: 0 to 1.25), and 0.90% (β-endorphin, 95% CI: 0.36 to 1.43) (Fig. 1 *A*, *Lower*). A third sEV biomarker, CD81, was present without CD9 or CD63 in only 3.47% of sEVs (*SI Appendix*, Fig. S1) and was therefore not followed further. Because CD63 is preferentially expressed on exosomes and CD9 on microvesicles (19), POMC may associate with both types of EVs, indicating that they are not solely present in the circulation as free, soluble molecules.

Since cross-reactivity can occur between POMC and its mature hormones (7), we next performed immunoblotting using Abs against either the first 150 amino acids of POMC or regions within ACTH or β-endorphin. Blotting of plasma sEVs and sEV-depleted plasma from adult volunteers revealed an immunoreactive band in both fractions with all three Abs, but corresponding only to the size of unprocessed POMC (~28 kDa) (Fig. 1*B*). The observed mass of POMC matched that of recombinant POMC produced in *Escherichia coli*, suggesting that POMC was not glycosylated (20). To exclude nonspecific binding, primary Abs were omitted (*SI Appendix*, Fig. S2). Surprisingly, no immunoreactive bands for sEVs were detected in the low molecular weight range (3 to 14 kDa), including the expected sizes of ACTH (~4.5 kDa) and β-endorphin (~3.5 kDa), even upon overexposure of the blot (Fig. 1*B*), indicating that bioactive peptides were either absent or present at levels below detection in sEVs. Consistently, POMC concentration measured by enzyme-linked immunosorbent assays (ELISA) were ~1,000-fold higher than those of the mature hormones ACTH and β-endorphin (Fig. 1*C*). These data suggest that unprocessed, full-length POMC is associated with plasma-derived sEVs. Subsequent studies therefore focused on POMC, given its high plasma concentration relative to its mature products.

To investigate the effects of intense physical exercise on POMC, blood was collected from 15 physically fit participants (10 males and 5 females, age range = 23 to 37) at three time points: before running (prerun), immediately after 50 min of running (postrun), and following a 30-min recovery period (postrun rest). Participants ran on a treadmill to achieve 76 to 96% of age-adjusted maximum heart rate, corresponding to a perceived exertion between 6 and 8 on a 10-point scale. Overall, size and concentration of sEVs did not change across the three time points (Fig. 1 *D* and *E* and *SI Appendix*, Fig. S3*A*). Similarly, plasma POMC concentration, measured by ELISA, did not change significantly after running (5.85 ± 0.68 and 5.36 ± 0.23 ng/mL, pre- and postrun, respectively, mean ± SEM, *P* = 0.55). Interestingly, there was an exercise-induced increase in sEV-associated POMC in individual runners. Magnitude of the increase varied by individual runner, from 1.3-fold to 11.7-fold, as analyzed by d-STORM (Fig. 1*F*; *P* < 0.001). At the postrun rest time point, the percentages of sEV-associated POMCs had partially reverted to prerun levels in most runners (Fig. 1*F*; *P*= 0.021), suggesting a reversible phenomenon. Permeabilizing the sEVs prior to d-STORM analysis did not change the POMC concentration (*SI Appendix*, Fig. S3*B*), indicating the absence of an additional intravesicular pool of POMC. ELISA confirmed the increase in POMC^+^ sEVs in postrun samples (Fig. 1*G*; 3.8-fold change, *P* = 0.002).

To further explore the association of POMC with sEVs after exercise, we analyzed plasma from three independent runners: the whole plasma, plasma depleted of larger particles by centrifugation at 10,000 × g (10K supernatant), and plasma depleted of sEVs by centrifugation at 100,000 × g (100K supernatant), using immunoblotting. Consistent with the exercise-induced increase in sEV-associated POMC, immunoblotting revealed a reduction of POMC in the 100K, but not in 10K, supernatant fraction, after running. This indicates that POMC in plasma exists either as a soluble form or bound to sEVs, and is not associated with larger particles (Fig. 1*H*). This latest series of experiments suggests that the form of POMC associated with EVs accounts for approximately one quarter of total POMC after running.

### Association between sEV Surface Receptors and POMC.

**Fig. 2.**
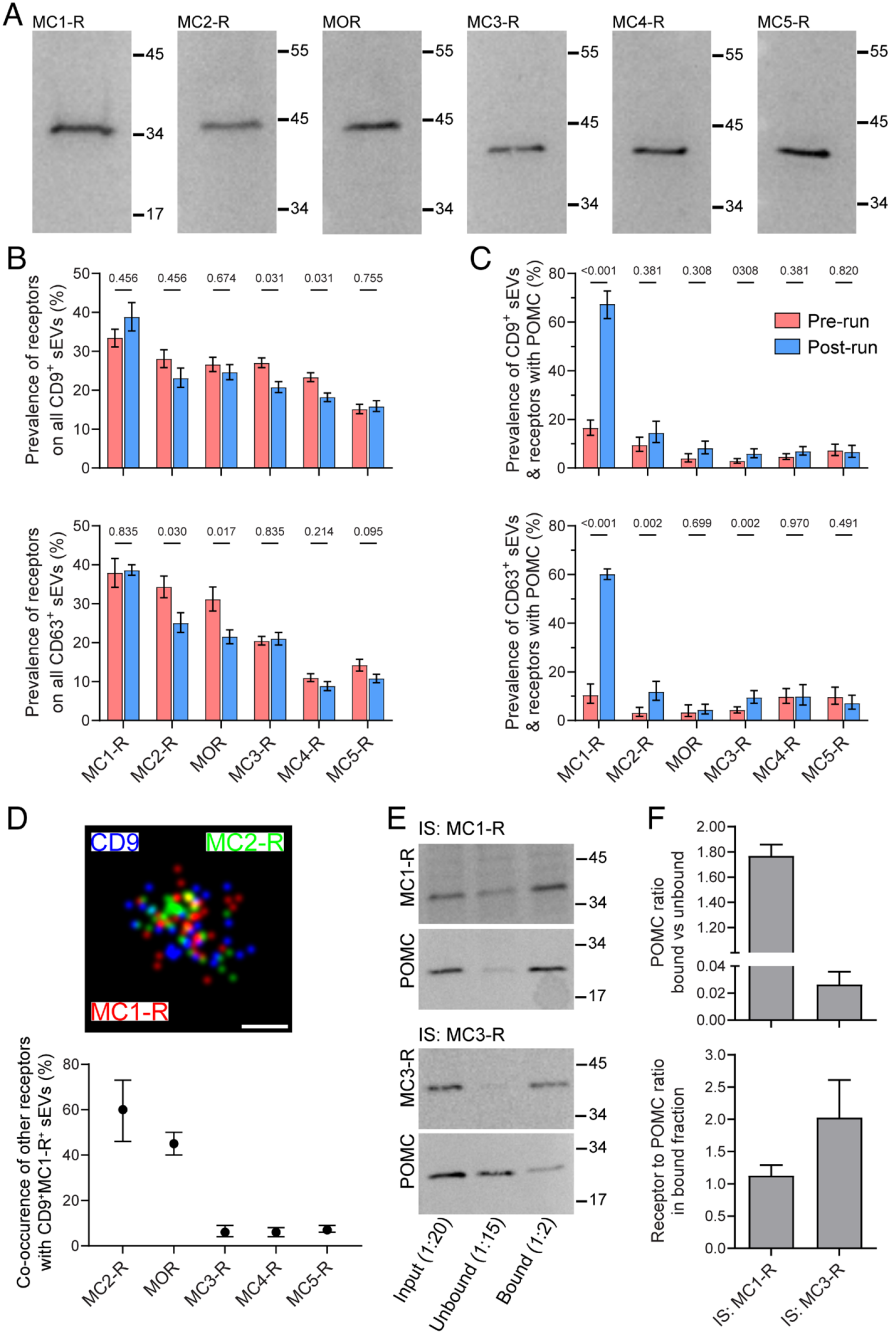
Plasma sEVs express receptors for POMC-derived hormones. (*A*) Enriched bloodborne sEVs from prerun plasma were analyzed by immunoblot for MC1-R, MC2-R, MOR, MC3-R, MC4-R, and MC5-R. Molecular mass markers (kDa) are indicated. (*B*) Prevalence of POMC-related receptors on all CD9^+^ (with or w/o CD63; *Upper*) or CD63^+^ (with or w/o CD9; *Lower*) sEVs isolated from the prerun and postrun plasma and quantified by d-STORM (n = 3 runners, mean ± 95% CI). (*C*) Association of POMC with CD9^+^ (*Upper*) and CD63^+^ (*Lower*) sEVs containing a given POMC-related hormone receptor, as indicated, from prerun and postrun plasma, were quantified by d-STORM (n = 3 runners, mean ± 95% CI). (*D*) To quantify co-occurrence between MC1-R and other receptors, sEVs were analyzed by d-STORM after triple immunolabeling for CD9, MC1-R, and another POMC-related hormone receptor. A representative d-STORM image is shown (*Upper*) and the percentage of sEVs coexpressing CD9 and MC1-R with another POMC-related hormone receptor was quantified (*Lower*, n = 3 runners, mean ± 95% CI). (*E*) sEVs from postrun plasma were solubilized and subjected to IS for MC1-R or MC3-R. Immunoblotting was performed on input, unbound, and bound fractions to detect POMC and the respective receptor. Representative blots are shown. Dilution factors used in loading were 1:20 for input, 1:15 for unbound, and 1:2 for bound fractions. (*F*) Quantification of IS experiments after correcting for dilution factors shown in *E*. The *Upper* panel shows the ratio of POMC in bound versus unbound fractions. The *Lower* panel shows the ratio of MC1-R or MC3-R to POMC in the bound fraction. Data are presented as mean ± SEM. (n = 3). *P*-values indicate comparisons between prerun and postrun in *B* and *C* (*Statistical Methods*).

Since POMC is not a membrane-associated protein (7), we hypothesized that plasma sEV-associated POMC binds to hormone receptors embedded in the sEV membrane. Immunoblotting of sEV lysates showed the presence of full-length melanocortin receptors 1, 3, 4, and 5 (MC1-R, MC3-R, MC4-R, and MC5-R), the mu -opioid receptor (MOR), and the glycosylated, lower-mobility form of the ACTH receptor (MC2-R) (21) (Fig. 2*A*). Imaging by d-STORM confirmed that these receptors localized to the surface of CD9^+^ and CD63^+^ sEVs (*SI Appendix*, Fig. S4*A*). Quantitative analysis showed that, prior to exercise, the prevalence of receptor-positive sEVs ranged from ~10 to 40%, with MC1-R^+^ consistently being the most abundant receptor population on both CD9^+^ and CD63^+^ sEVs (Fig. 2*B*). Following running, most receptor levels remained unchanged; however, although effect sizes were small, in CD9^+^ sEVs both MC3-R and MC4-R were less prevalent after running (*P* = 0.031 for each), while in CD63^+^ sEVs MC2-R and MOR were less prevalent after running (*P* = 0.030 and *P* = 0.017, respectively) (Fig. 2*B*).

We next examined the localization of POMC on the surface of sEVs carrying POMC-related receptors using d-STORM. Prior to exercise, POMC was detected on ~15% of CD9^+^ sEVs and ~10% of CD63^+^ sEVs harboring one or more POMC-associated receptors (Fig. 2*C* and *SI Appendix*, Fig. S5). Following exercise, the proportion of POMC^+^MC1-R^+^ sEVs increased markedly, by 4.1-fold (to 67%) for CD9^+^ sEVs and 5.8-fold (to 60%) for CD63^+^ sEVs. More modest postexercise increases were also observed for POMC association with MC2-R^+^CD63^+^ sEVs (2.2-fold) and MC3-R^+^CD63^+^ sEVs (2.1-fold). To rule out potential nonspecific binding of POMC to sEVs, we incubated those derived from SW480 cells and human dermal fibroblasts (HDFs) with recombinant POMC (rPOMC) at physiological pH (7.4). SW480-derived sEVs, which express MC1-R, exhibited modest rPOMC binding (~15%), indicating that POMC engages MC1-R on the sEV surface. In contrast, HDF-derived sEVs, which lack MC1-R, showed no detectable rPOMC binding (*SI Appendix*, Fig. S4*B*).

Interpretation of POMC association with sEVs relative to receptors was complicated by preferential co-occurrence of receptors on sEVs (chi-square test *P* < 0.001; Fig. 2*D*). For CD9^+^-MC1-R^+^ plasma sEVs, 60% co-occurred with MC2-R and 42% with MOR, whereas CD9^+^-MC1-R^+^ plasma sEV co-occurrence with MC3-R, MC4-R, and MC5-R was <10%, in congruence with random expectations (chi-square test *P* > 0.05). Patterns were similar for CD63^+^ sEVs. To further investigate which sEV-associated receptors may bind POMC in vivo, we did magnetic beads-based immunoisolation (IS) on postrun plasma sEV lysates. For each receptor, the input, unbound, and bound fractions are presented. Measurable POMC was recovered in the bound fractions of MC1-R and MC3-R (Fig. 2*E*), but only trace amounts in the bound fractions of MC2-R, MC4-R, MC5-R, or MOR (*SI Appendix*, Fig. S4*C*), indicating preferential association of POMC with MC1-R and, to a lesser extent, MC3-R. This is consistent with the smaller increases in POMC association with MC2-R, MC4-R, MC5-R, or MOR observed by d-STORM after exercise (Fig. 2*C*). Quantification of the IS experiments, corrected for the dilution factors used in loading (input 1:20, unbound 1:15, bound 1:2), showed that the ratio of POMC in bound versus unbound fractions was highest for MC1-R, with a smaller but detectable association for MC3-R (Fig. 2 *F*, *Upper panel*). When normalized to POMC, the amount of receptor in the bound fraction confirmed that MC1-R was the predominant binding partner for POMC compared with MC3-R (Fig. 2 *F*, *Lower panel*). Importantly, a negative control using Protein G beads without primary Ab confirmed that receptors were not pulled down in the absence of targeted IS (*SI Appendix*, Fig. S4*D*).

### Cell Source of Plasma sEVs with MCRs and MOR and Association with POMC.

**Fig. 3.**
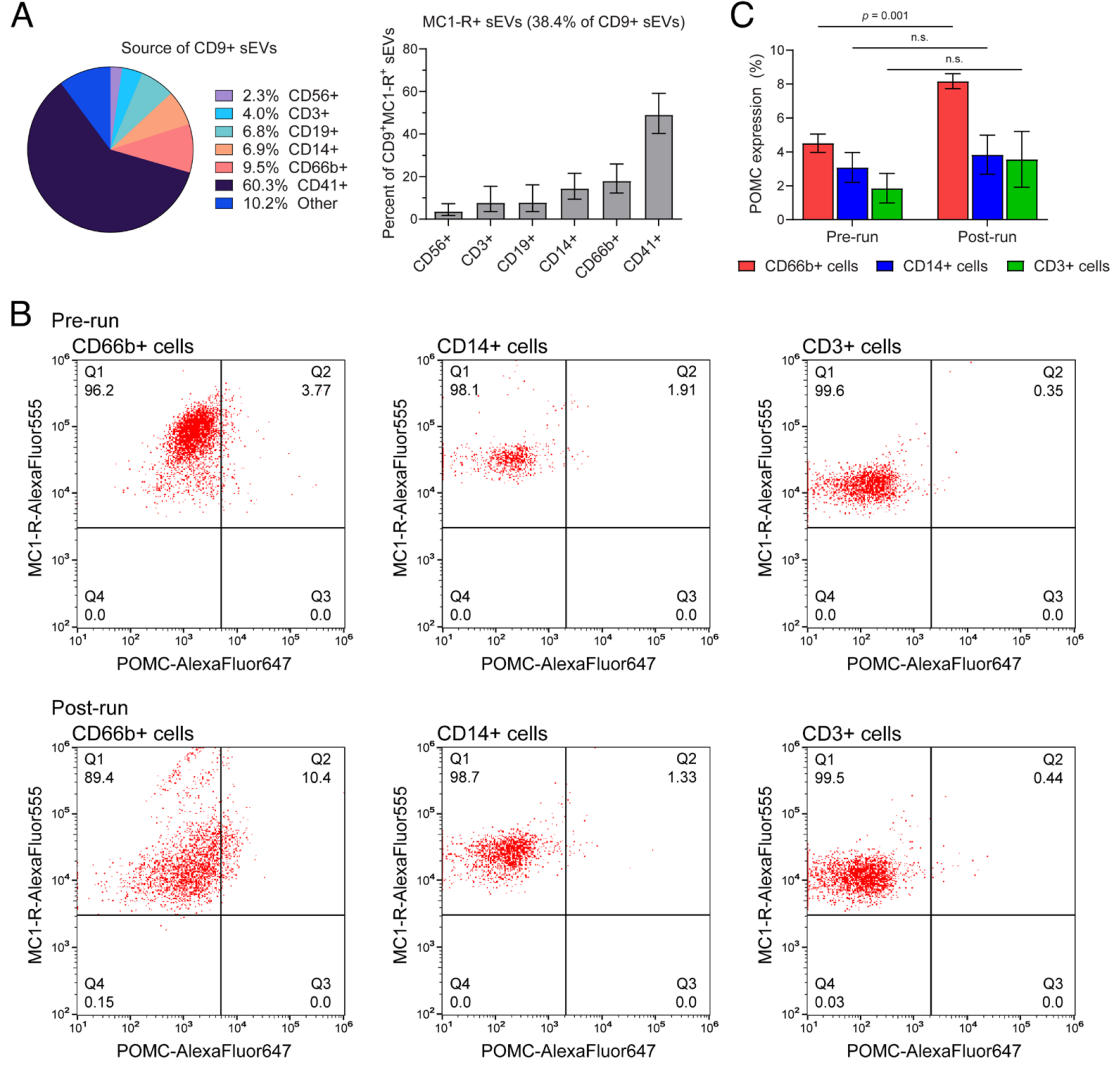
sEVs expressing hormone receptors derived from leukocytes and platelets. (*A*) sEVs derived from prerun plasma were immunolabeled for CD9, MC1-R, and markers for natural killer cells (CD56), T cells (CD3), B cells (CD19), monocytes (CD14), granulocytes (CD66b), or platelets/megakaryocytes (CD41) prior to analysis by d-STORM. The percentage of CD9^+^ sEVs derived from various blood cell types is shown (*Left*), and among CD9^+^ sEVs, the association of MC1-R with subpopulations derived from leukocytes or platelets was quantified (mean ± SEM, n = 3). (*B*) Leukocytes isolated from prerun and postrun blood were immunolabeled for MC1-R, POMC, and CD markers specific to granulocytes (CD66b), monocytes (CD14), or T cells (CD3) and analyzed by flow cytometry. Representative flow cytometry plots show the coexpression of MC1-R and POMC in each cell subset. Percentages are displayed in the quadrants. (*C*) Quantification of POMC expression in each leukocyte subset (CD66b^+^ granulocytes, CD14^+^ monocytes, and CD3^+^ T cells) from prerun and postrun blood samples. Data are presented as mean ± SEM from five independent runners (n = 5). *P*-values are indicated.

To investigate the cell source of plasma sEVs with MC1-R, we coimmunolabeled sEVs for CD9 and specific markers for blood cells using d-STORM. Blood cell-specific markers were absent in 10.2% of CD9^+^ sEVs. The remaining 89.8% of plasma-derived CD9^+^ sEVs consisted of 2.3% CD56^+^ (likely natural killer cells), 4.0% CD3^+^ (likely T cells), 6.8% CD19^+^ (likely B cells), 6.9% CD14^+^ (likely monocytes/macrophages), 9.5% CD66b^+^ (likely granulocytes), and 60.3% CD41^+^ (likely platelets or megakaryocytes; Fig. 3*A*), in agreement with other studies (22–24) that found platelets and megakaryocytes are the predominant cell source of plasma sEVs. Of the ~38% CD9^+^ sEVs that were MC1-R^+^, 49.0% were CD41^+^, with CD14^+^ (14.3%), CD66b^+^ (17.9%), and CD3^+^ (7.6%) all overrepresented relative to their prevalence (*P* < 0.001; Fig. 3*A*). Therefore, prerun, 18.6% of CD9^+^ sEVs in plasma were MC1-R^+^ and derived from platelets or megakaryocytes, with the remaining 19.4% CD9^+^ sEVs with MC1-R^+^ from leukocytes or unknown cellular sources (Fig. 3*A*). Since granulocytes, T cells, and monocytes have MCR on their membranes, we measured POMC association with cells before and after running using flow cytometry. POMC association with granulocytes almost doubled after running (*p*=0.001); increases in POMC association with T cells and monocytes were smaller in magnitude, but not statistically supported (*P* > 0.05, Fig. 3 *B* and *C*).

### sEV-Bound POMC Crosses Endothelial Barriers and Stimulates Melanin.

**Fig. 4.**
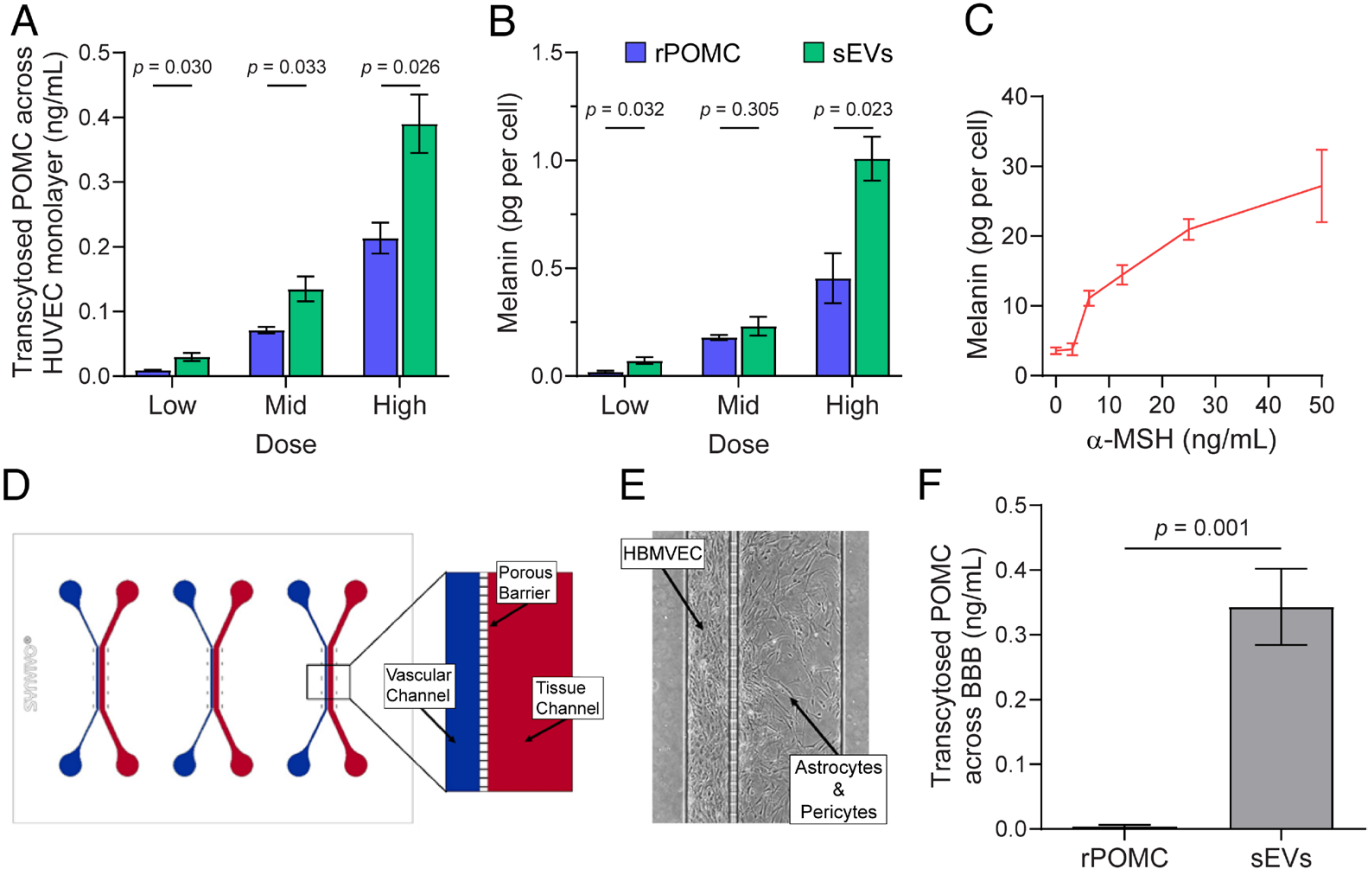
Passage of POMC through blood–brain barrier or endothelial layer and stimulation of melanin production. (*A*) Passage across HUVEC monolayers. HUVECs were seeded in Transwell inserts and cocultured with B16-F10 cells in the lower chamber. rPOMC (Low = 0.5, Mid = 1.0, High = 3.0 ng/mL) or postrun sEVs (Low = 2 × 10^9^, Mid = 5 × 10^9^, High = 1 × 10^10^ particles) were added to the upper chamber. POMC concentration in the lower chamber was measured after 4 h by ELISA (mean ± SEM, n = 3). (*B*) Melanin production by B16-F10 cells following transport assays in panel *A*. Low, mid-range, and high doses of rPOMC or sEVs were defined as in panel *A*. Melanin content was quantified after 48 h by absorbance at 490 nm (mean ± SEM, n = 3). (*C*) B16-F10 cells were incubated for 48 h with increasing concentrations of α-MSH as positive control. Melanin content was quantified by absorbance at 490 nm (mean ± SEM, n = 3-18 independent experiments). (*D*–*F*) Transcytosis across an in vitro BBB microfluidic model. Schematic of the SynBBB™ 3-PLEX microfluidic chips model. (*D*) The device consists of three parallel experimental units, each with a 200-μm vascular channel (blue) and a 500-μm tissue channel (red) separated by a 3 × 3 μm porous barrier. Representative phase-contrast image of a fully functionalized human BBB model with vascular lumenization of primary human brain microvascular endothelial cells and cocultured with astrocytes and pericytes, establishing a physiologically relevant barrier suitable for compound dosing. (*E*) Experimentally, SynBBB chips were exposed for 4 h to rPOMC (3 ng/mL) or plasma-derived postrun sEVs (1 × 10^10^ particles). (*F*) POMC in the tissue-channel effluent was quantified by ELISA (mean ± SEM, n = 4). *P*-values are indicated. The cartoon and micrograph presented in panels *D* and *E* were provided by Synvivo.

As G-protein-coupled receptors, MC1-R and other MC-Rs cannot initiate signaling pathways within sEVs. However, sEV-bound POMC may become available to elicit functional responses by binding to MC1-R or MC3-R on cells. To address these issues, we first determined how much POMC is carried by sEVs. We quantified POMC levels by ELISA across increasing particle concentrations and found a proportional increase in detectable POMC: 0.48 ± 0.08 ng/mL at 2 × 10^9^ particles, 1.60 ± 0.15 ng/mL at 5 × 10^9^ particles, and 3.57 ± 0.32 ng/mL at 1 × 10^10^ particles. We next asked whether POMC associated with sEVs can pass through an endothelial layer and stimulate a cellular response. To this end, we employed a Transwell filter model in which human umbilical vein endothelial cells (HUVEC) were seeded in the upper chamber and cocultured with B16-F10 melanoma cells expressing MC1-R (25) in the lower chamber. The addition of postrun sEVs or rPOMC as a control at varying concentrations to the upper chamber led to detectable POMC in the lower chamber after 4 h of incubation, as measured by ELISA (Fig. 4*A*). Under these conditions, sEV-associated POMC reached approximately twofold higher concentrations than rPOMC in the lower chamber, independent of the POMC dose applied, suggesting that sEV-associated POMC can pass through a simple endothelial cell monolayer. Then, we evaluated melanin production by B16-F10 cells exposed to POMC after 48 h, as determined by absorbance at 490 nm (26). Interestingly, melanin production increased significantly in a POMC dose-dependent manner (Fig. 4*B*). As a positive control, melanoma cells were treated with α-MSH, which similarly induced melanin production (Fig. 4*C*), consistent with previous observation in mouse and human melanoma cells (10). In these experiments, we used 10^9^–10^10^ particles per milliliter, as determined by nanoparticle tracking analysis, a concentration range within the physiological levels reported for human plasma (2, 3).

Because the BBB is more complex than a simple HUVEC monolayer (27, 28), we employed commercial SynBBB™ 3-PLEX microfluidic chips in which brain microvascular endothelial cells, astrocytes, and pericytes were cocultured on a porous substrate, thereby more closely mimicking the three-dimensional (3D) physiological characteristics of the BBB (29, 30) (Fig. 4 *D* and *E*). Using this advanced model, we compared the transcytosis of rPOMC (3 ng/mL) with that of postrun sEVs (1 × 10^10^ particles). The sEV dose was chosen to provide a POMC amount approximately equivalent to 3 ng/mL (see above), enabling a direct comparison between the two inputs. After 4 h of incubation, POMC was detectable by ELISA in the effluent only when sEVs were applied, but not when rPOMC was added (Fig. 4*F*), indicating that sEV association facilitates POMC transcytosis across the BBB.

### Blood pH as a Modulator of the Association between POMC and sEV-Based MC Receptors.

**Fig. 5.**
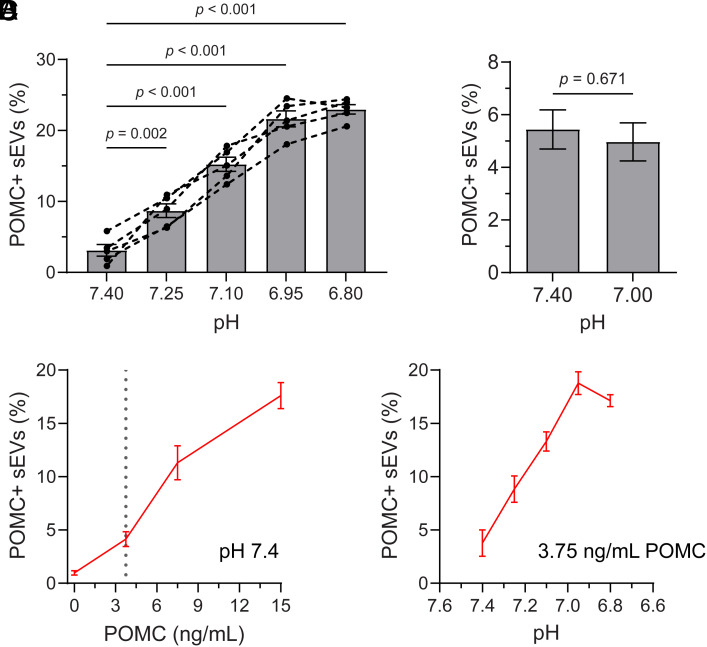
Effect of pH on binding of POMC to sEVs. (*A*) The pH of the total plasma from prerun samples was adjusted as indicated (7.40 to 6.80) with PBS at pH ranging from 3.8 to 6.4 and incubated for 50 min. sEVs were analyzed by d-STORM to determine the percentage of POMC^+^ sEVs. Each set of connected dots represents data from an individual runner (n = 5), with mean ± SEM shown. (*B*) To test the rate of dissociation for sEV-bound POMC, sEVs enriched from prerun plasma were incubated at two different pH (7.4 and 7.0) for 50 min and analyzed by d-STORM. The percentage of POMC^+^ sEVs are shown (mean ± SEM, n = 3). (*C* and *D*) sEVs derived from B16-F10 cells were incubated with either an increasing concentration of rPOMC (0 to 150 ng/mL) at pH = 7.4 (*C*) or decreasing pH (7.4 to 6.8) at 3.75 ng/mL POMC (indicated by the dotted line in *C*) (*D*) for 50 min. In both cases, the percentage of POMC^+^ sEVs was determined by d-STORM (mean ± SEM, n = 3). *P*-values are indicated.

We hypothesized that exercise-induced pH changes in the blood could influence the association between POMC and sEV receptors. A pH range of 7.4 to 6.8 is consistent with normal physiology during exercise in trained and untrained exercisers’ blood (31); more acidic pH is associated with higher workload (32). When pH was experimentally lowered in prerun plasma collected from five participants, POMC association with sEVs increased incrementally from 2.6 to 23.7% (Fig. 5*A*). To assess whether already bound POMC disassociates from the sEVs, we incubated enriched sEVs at two different pH (7.4 and 7.0) for 50 min. Results indicate no significant disassociation of POMC from sEVs at either pH level (Fig. 5*B*). Increasing the concentration of rPOMC at pH 7.4 resulted in a concentration-dependent increase in the percentage of POMC^+^ EVs (Fig. 5*C*). When POMC concentration was held constant and pH was lowered from 7.4 to 7.0, the percentage of POMC^+^ EVs increased (Fig. 5*D*). The proportion of POMC^+^ EVs at pH 7.0 was comparable to that observed at the highest rPOMC concentration tested at pH 7.4 (Fig. 5 *C* and *D*), indicating that lowering pH can enhance POMC–EV association to a level similar to high ligand availability.

**Fig. 6.**
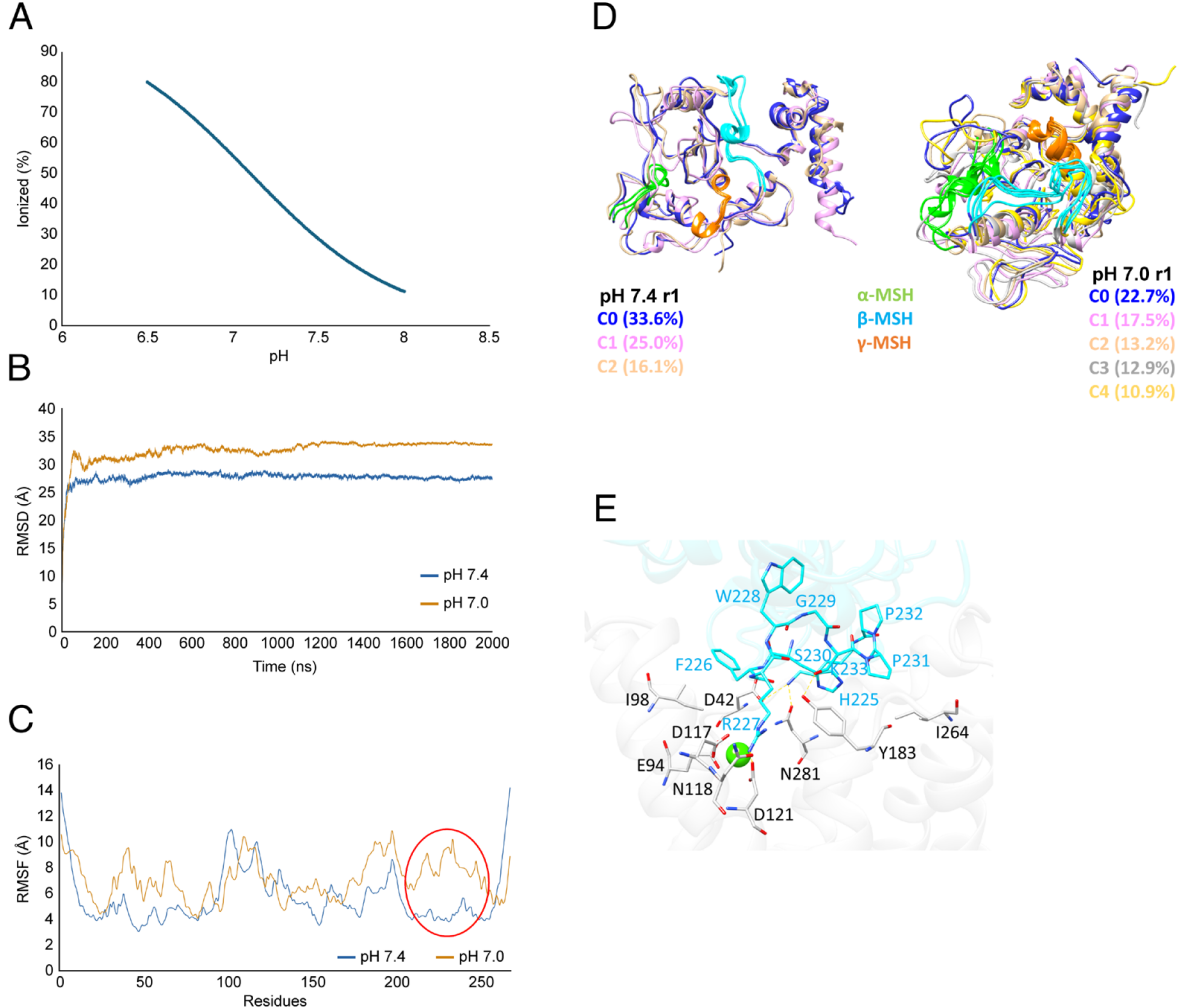
Molecular modeling of POMC at different pH levels. (*A*) Percentage of ionized His143 in the pH range of 6.5 to 8.0, computed using the Henderson–Hasselbalch equation and based on a predicted pK of 7.1. (*B*) RMSD profiles for a 2 μs MD simulation performed at pH 7.0 and 7.4. The plotted profiles correspond to the RMSD means from two replicates. (*C*) RMSF profiles for the same MD simulations. The red circle highlights the segment corresponding to β-MSH. (*D*) Representative structure of the hormone-receptor cluster at pH 7.4 (neutral state) and pH 7.0 (ionized state). Clusters represent conformations present for at least 10% of the simulation time and were used for protein–protein docking calculations. Structures extracted from the first replicates are displayed. (*E*) Main interactions stabilizing the complex between the β-MSH segment of POMC (azure) and the MC1-R binding site (white). The green sphere represents the Ca^++^ ion, which is essential for MC1-R activity. The POMC structure corresponds to a common conformation from the first simulation run at pH 7.0.

To better understand how pH modulates the interaction between POMC and its potential receptors, we pursued molecular modeling. Based on 2 µs molecular dynamics (MD) simulations to optimize and stabilize overall folding of the POMC structure (retrieved from Alphafold Protein Structure Database), an analysis of the ionizable residues (as performed by the H++ webserver) suggested that His143, a histidine within the α-MSH domain of POMC, becomes increasingly likely to be protonated during acidification between pH 8.0 and 6.5 (Fig. 6*A*). Protonation induced rapid conformational change (<100 ns) followed by a relatively stable conformation (Fig. 6*B*), confirmed by Ramachandran plots (*SI Appendix*, Fig. S6*A*). Subsequent analysis of the stable structures suggested that RMS fluctuations (RMSF) were similar between pH 7.4 and pH 7.0 in the POMC domains corresponding to γ-MSH and α-MSH, while the β-MSH domain had increased flexibility and mobility at pH 7.0 compared to pH 7.4 (Fig. 6*C*). Analysis of the per-residue solvent accessible surface area (*SI Appendix*, Fig. S6*B*) revealed that at pH 7.0 the POMC fragment related to β-MSH is more exposed to the solvent and, therefore, it might be more prone to interact with MCRs.

Cluster analysis of the most common POMC conformations in the two ionization states showed that the β-MSH segment assumes a more internal position at pH 7.4, shifting to a more externalized conformation in the form of a flexible loop at pH 7.0 (Fig. 6*D*). In protein–protein docking simulations, the β-MSH segment of one of the POMC conformations at pH 7.0, labeled C3, elicited the key interaction with MC1-R by arranging the arginine side chain like the bound hormone α-MSH (Fig. 6*D*). The corresponding POMC/MC1-R complex involved interactions among the neighboring POMC aryl and alkyl side chains, which are hydrophobic, and π–π stacking interactions with the surrounding apolar MC1-R residues. The overall POMC/MC1-R complex was stabilized by interactions between residues that did not belong to POMC β-MSH or the MC1-R binding cavity, involving, for example, the ionic interactions between 1) Lys255 of POMC and Asp184 of MC1-R, 2) Glu30 of POMC and Arg109 of MC1-R, 3) Arg236 of POMC and Glu269 of MC1-R and 4) Glu165 of POMC and Lys278 of MC1-R (Fig. 6*E*).

In the simulations, MC3-R, MC4-R, and MC5-R mirrored MC1-R in interacting with the β-MSH segment of POMC at pH 7.0 (*SI Appendix*, Fig. S6*C*), while MC2-R (ACTHR) did not interact with POMC at any pH. The key ion-pair between the arginine residue of the HFRW binding motif and the MCR aspartate residue was Asp 117 in MC1-R, Asp 121 in MC3-R, Asp126 in MC4-R and Asp119 in MC5-R. None of the POMC conformations at pH 7.4 yielded comparable binding complexes with the receptors. Since the interaction with MOR requires the free N terminus of β-endorphin, the corresponding fragment of POMC is not able to assume conformations conducive to interaction with MOR in the full-length protein (in which the N terminus is masked by the peptide bond) at any pH.

**Fig. 7.**
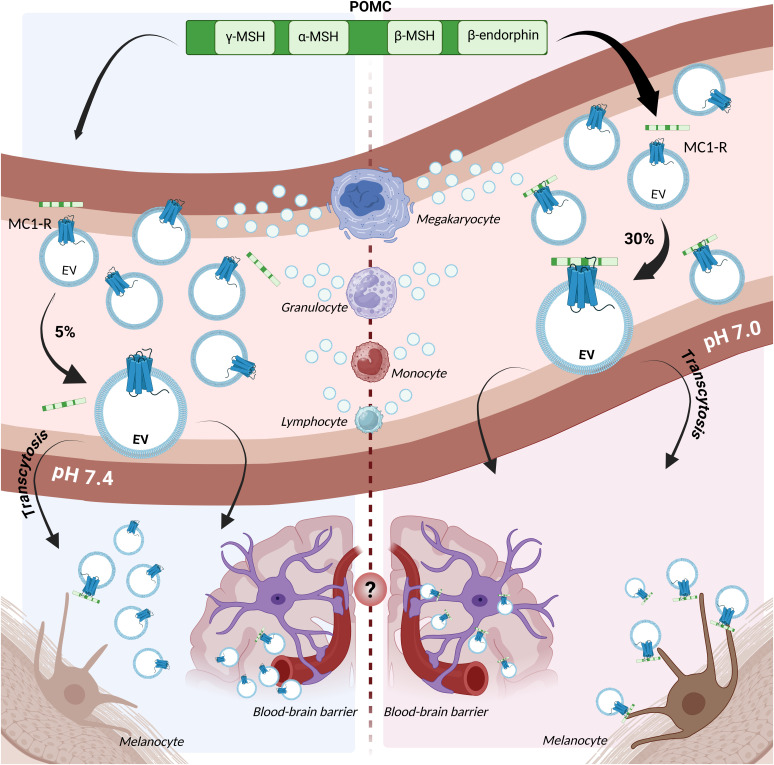
Summary of findings and potential downstream effects. Illustration of a blood vessel with sEVs carrying the melanocortin receptor 1 (MC1-R). Most bloodborne MC-1R^+^ sEVs are produced by megakaryocytes/platelets and leukocytes. Changes in POMC conformation from pH 7.4 (*Left*) to pH 7.0 (*Right*) favor binding to MC-1R^+^ sEVs and MC-1R^+^ cells such as melanocytes, where binding induces melanin production. POMC association with sEVs in the blood may facilitate passage through the blood–brain barrier and endothelium (indicated by “?”) (33). Created in BioRender: https://BioRender.com/s78u768.

In summary, the results suggest that the temporary blood acidification induced by vigorous exercise increases affinity between MC-Rs, particularly MC1-R, and POMC (Fig. 7). Most circulating sEVs are produced by blood cells, particularly megakaryocytes/platelets and leukocytes, and a subset of the sEVs carry MC1-R on their membrane. Therefore, blood acidification increases association between circulating POMC and sEVs or MC1-R^+^ leukocytes. Moreover, sEV-bound POMC travels more easily through the endothelium and the blood–brain barrier and it is, like unbound POMC, available to invoke a functional response by binding with MC1-R on cells.

## Discussion

In this study, we investigated the presence and functional dynamics of POMC in the plasma of healthy adults and demonstrated that: i) unprocessed, full-length POMC is present at substantially higher concentrations than its processed peptide hormones; ii) a fraction of circulating POMC associates with sEVs through binding to POMC-related receptors; iii) sEV-bound POMC increases ~fourfold following intense physical exercise; iv) exercise-induced blood acidification promotes a conformational change in the POMC structure, enhancing its interaction with receptors on the sEV surface as well as on leukocytes; and v) sEV association facilitates POMC transport across the BBB and an endothelial monolayer in vitro compared to soluble POMC, and preserves the ability to initiate a cellular response as shown by melanin production assay.

As a circulating prohormone, POMC has been a conundrum since its discovery in the mid-1970s (7). Studies capable of distinguishing between unprocessed POMC and POMC-derived mature hormones, ACTH and α-MSH, have found constitutive secretion of POMC (11), whereas secretion of mature hormone is linked to the regulatory pathway (34, 35). Accordingly, POMC concentrations are consistently higher than those of its mature hormones, both before and after exercise. This pattern aligns with previous reports of elevated full-length POMC relative to its derivatives in human skin (10) and cerebrospinal fluid (36). As we found no evidence for exercise-induced increases in secretion, we propose that the circulating POMC in this study was released primarily via the constitutive secretory pathway (11). Although we identified the cellular origin of the circulating sEVs, the specific source of POMC carried by these sEVs was not explored here. Previous studies have reported POMC production in many tissues, including the hypothalamus, pituitary, lymphocytes, macrophages, and skin (7, 37–39).

Previous work had shown that, despite being a prohormone, freely circulating POMC can induce melanin production in human epidermal melanocytes (9). Our study extends this finding by demonstrating that POMC associated with sEVs can also elicit a functional response. Although the binding kinetics of POMC to MC1-R remain unclear, our experiments suggest that association occurs rapidly in response to pH changes, whereas dissociation is considerably slower.

Our observation that POMC associates with sEVs was unexpected but aligns with a proteomic report associating POMC with sEVs in the luminal fluid of the uterus of pregnant ovines (40). A similar interaction could involve surface receptors on EV membranes and cytokines (41). The inducible association between sEV membrane proteins and the prohormone POMC reported here adds a mechanism by which circulating sEVs intersect with the endocrine system (2). To date, studies examining the interplay between EVs and the endocrine system have not implicated EVs in hormone transport; rather, they have focused on endocrine gland production of EVs, synergistic and antagonistic effects of EVs and hormones on target cells, hormonal regulation of EV production, and EV-mediated regulation of hormone production (2).

The presence of MC receptors in sEV membranes derived by leukocytes is predictable from their expression on parent cells, including monocytes/macrophages, granulocytes, B lymphocytes, NK cells, and CD4^+^ T cells, all of which express MC1-R (42). Additionally, MC1-R and MOR proteins have been reported in EVs from human mesenchymal stem cells (43) and T cells (44, 45). The mRNA for MC2-R and MC3-R have been detected in mesenchymal stromal cell-derived EVs (46). This study reports the observation of MC2-R, MC3-R, MC4-R, and MC5-R proteins present in EV membranes. As the main source of sEV populations carrying MC receptors and MOR, CD41^+^, CD66b^+^, and CD14^+^ blood cells have the potential to impose considerable regulatory control on POMC availability and transport. However, we did not observe substantial changes in the prevalence of MC receptors on circulating sEVs in response to exercise. The experimental evidence suggests that POMC preferentially binds to MC1-R, and somewhat with MC3-R, whereas molecular modeling predicts potential interaction of POMC with MC4-R and MC5-R as well. Further studies are needed to reconcile this discrepancy.

The full physiological implications of a pH-driven shift from soluble to sEV-bound POMC form will require additional study. Previous work on carboxypeptidase E (CPE) demonstrated that association with EVs was required for CPE to induce proliferation and growth of hepatocellular carcinoma cells (47, 48). Our results indicate that association with sEVs enhances POMC transcytosis across BBB in vitro and increases its ability to induce melanin production. Consistent with previous reports that HUVEC monolayers do not fully recapitulate the barrier properties of brain endothelial cells in the presence of other supporting cells (27, 28), transcytosis of rPOMC through a simple endothelial monolayer does not reflect the in vivo situation. Accordingly, the transcytosis of POMC associated with sEVs in SynBBB™ 3-PLEX microfluidic chips represents a physiologically relevant mechanism, since the EV-bound form accounts for only a fraction of total blood POMC.

Several alternative and nonexclusive hypotheses may explain how sEV-associated POMC interacts with target cells and affect downstream signaling: i) POMC could be processed in the extracellular space, as supported by a report in mouse cells showing that trypsin-like enzymes can convert POMC to ACTH (49), with the resulting bioactive hormones binding to cell receptors; ii) POMC^+^ sEVs may be internalized by endocytosis and, through the retrograde trafficking, reach POMC convertase enzymes in the trans-Golgi network (2, 50), enabling enzymatic processing; iii) sEV-associated POMC may engage receptors at the cell surface (4) through a POMC domain not involved in the binding with the sEV-embedded receptor; and/or iv) an unidentified trigger near the target cell may accelerate the rate of POMC dissociation from sEVs, increasing cell receptor binding.

### Limitations and Future Directions.

Accurate measurements of blood pH remain challenging (51), and the effects of different types of exercise or stress on blood pH need to be clarified. Given the frequent conflation of β-endorphin and POMC in exercise studies, as well as inconsistent data on changes in sEVs during exercise (reviewed in ref. 14), no definite conclusions can yet be drawn regarding the potential relationship between pH-dependent POMC receptor binding and the dose-dependent benefits of exercise (52, 53). Moreover, our results from healthy, fit runners may not extend to other populations, as previous reports have shown that EV-associated miRNA differs between sedentary and fit older men (52).

Impact of pH on POMC binding to receptors beyond the melanocortin receptors, such as neuronal and endocrine receptors (54), is unknown. The incompleteness of the POMC literature regarding production, circulation, binding affinities, and processing is a symptom of high complexity. For example, beyond the cross-reactivity between Abs for POMC and its mature hormones already discussed, membrane interactions can induce POMC to shift into alpha-helix structures (55) and POMC molecules form dimers and multimers at biologically relevant pH (56). Moreover, this study focused on sEVs. The potential for interactions with large EVs was only briefly addressed (Fig. 1*D*), although it yielded no evidence for large EV interaction with POMC.

The finding of a separate mechanism by which sEVs associate with POMC in the blood—raises numerous questions for future investigation. For instance, sEV association may inform more accurate pharmacokinetic models of drugs that target with MC-Rs or opioid receptors (57), such as Bremelanotide, Setmelanotide, morphine/methadone/fentanyl, and Eluxadoline (37). Additionally, POMC binding to MC1-R on leukocytes can invoke an anti-inflammatory response (37), which may contribute to the systemic effects of exercise (58).

## Materials and Methods

### Subjects and IRB.

Fifteen individuals (n = 15; male and female; age 22 to 35 y) who regularly participated in endurance training with a body mass index (BMI) in the range of 18.5 to 24.9 were recruited for this study after signing informed consent. Participants engaged in a 50-min running session at a steady intensity, maintaining 76 to 96% of their age-adjusted maximum heart rate. The study protocol was approved by the Touro University Nevada Institutional Review Board (TUN IRB#000225).

### Isolation and Characterization of sEVs.

Blood samples (5 mL) were collected from participants at the three time points described above. Plasma was prepared by centrifuging at 2,000 × g for 10 min at room temperature (RT). To enrich sEVs, plasma was diluted 1:1 with phosphate-buffered saline (PBS) and sequentially centrifuged at 2,000 × g and 10,000 × g for 30 min each at 4 °C to remove cellular debris and larger particles. The resultant supernatant was subjected to ultracentrifugation at 100,000 × g for 60 min at 4 °C using a Beckman Coulter Optima XE-90 ultracentrifuge equipped with an SW41Ti rotor. The sEV pellet was resuspended in PBS, and ultracentrifugation was repeated under the same conditions. The final sEV pellet was resuspended in 100 µL of PBS. sEV size and concentration were determined using nanoparticle tracking analysis (NTA) on the ZetaView system (Particle Metrix GmbH) as described (59). Measurements were performed in scatter mode using a 488 nm laser, capturing videos at 30 frames per second across 11 different positions along the z-axis, with 2-s videos per position. Instrument parameters were set to a camera sensitivity (gain) of 10 and a minimum trace length of 15. sEVs were diluted in sterile filtered PBS prior to injection into the instrument. For each sample, three technical replicates were recorded. sEVs were characterized by immunoblotting and d-STORM according to the MISEV 2023 guidelines (16). The process of lysing blood sEVs interfered with detection of the mature hormones; therefore, concentrations were tested on samples where sEVs were not lysed. The plasma supernatant was stored at −80 °C for downstream analyses. NTA confirmed that sEVs isolated from the plasma of healthy volunteers fell within the expected sEV size range (~100 to 150 nm), and that permeabilization did not alter size or concentration (*SI Appendix*, Fig. S1*A*). Immunoblotting showed sEV markers CD9, CD63, CD81, and Alix, while calnexin (CNX) was absent, confirming sample purity (*SI Appendix*, Fig. S1*B*). Using d-STORM, we next evaluated marker expression under nonpermeabilized and permeabilized conditions (*SI Appendix*, Fig. S1*C*). Surface tetraspanins CD9, CD63, and CD81 were readily detected without permeabilization, while the intravesicular protein Alix became detectable only after permeabilization, confirming membrane integrity. CNX remained absent in both conditions, excluding cellular contamination. Quantification revealed the relative distribution of sEVs positive for one or more of these markers: CD9, CD63, CD81, and Alix, while CNX was consistently undetectable (*SI Appendix*, Fig. S1*C*). These results confirm that the preparations were intact, pure sEVs, and suitable for subsequent analyses of POMC and its peptide derivatives.

### d-STORM.

Enriched sEVs were immunolabeled and analyzed using the EV Profiler Kit (#EV-MAN-1.0, ONI) and d-STORM. sEVs were immobilized on microfluidic chips provided with the commercial kit and fixed with F1 solution for 10 min. For permeabilized samples, sEVs were treated with 0.01% Triton X-100 for 10 min (59). sEVs were then incubated with fluorescently labeled Abs prepared in permeabilization buffer or PBS, as detailed (*SI Appendix*, Table S1) for 50 min at RT. Following incubation, samples were postfixed with F1 solution and imaged in freshly prepared d-STORM imaging buffer. Imaging was performed using the ONI Nanoimager S microscope equipped with four laser lines (405, 488, 561, and 640 nm). Each field of view was recorded using a 100× oil immersion objective (NA 1.4), and 5,000 frames per channel were captured at an exposure time of 30 ms per frame. Drift correction and localization precision analysis were performed in real time using the ONI CODI online analysis platform. Subpopulation clustering of sEVs expressing one, two, or three markers was performed using density-based spatial clustering of applications with noise (DBSCAN) algorithms with localization filtering parameters set according to ONI recommendations. A minimum of 3,500 sEVs were counted per experiment.

### IS.

sEVs were lysed in ice-cold buffer I (0.5% Triton X-100, 150 mM NaCl, 50 mM Tris-HCl, pH 7.4) containing protease inhibitor cocktail (Set III, #539134, Calbiochem, Merck). Lysates were incubated with specific Abs directed against MC1-R, MC2-R, mu Opioid, MC3-R, MC4-R, MC5-R, or POMC, followed by Protein G-conjugated magnetic beads (#130-071-101, Miltenyi Biotec) as per manufacturer instructions. Samples were processed through µ Columns (#130-042-701, Miltenyi Biotec) mounted on a magnetic stand. Postwashing, bound fractions were eluted with preheated Tricine-SDS buffer and analyzed by immunoblotting.

### Immunoblotting.

sEVs, plasma supernatant, or recombinant protein samples were diluted in Tricine-SDS sample buffer (#LC1676, Fisher Scientific) supplemented with β-mercaptoethanol, except for nonreducing blots targeting CD9, CD63, and CD81. Samples were heated to 95 °C for 5 min, and proteins were resolved on 16% Tricine precast gels (#EC66955BOX, Fisher Scientific) alongside a prestained molecular weight ladder (#LC5925, Fisher Scientific). Proteins were transferred to PVDF membranes (#PI22860, Fisher Scientific) for 40 min at 4 °C.

### ELISA.

POMC, ACTH, and β-endorphin levels in plasma and POMC in enriched sEVs were quantified using the ELISA kits (#BSKH62208, #BSKH60373, and BSKH60909, respectively; Bioss Inc. MA) according to the manufacturer’s protocol.

### Flow Cytometry.

Labeled cells were analyzed using the CytoFlex flow cytometer (Beckman Coulter). Gain settings for all detectors were standardized across experiments. At least 100,000 events per sample were recorded, and FlowJo software (version 10.7.2, BD Life Sciences) was used for data analysis to identify specific cell populations.

### Blood–Brain Barrier Transcytosis Assay.

SynBBB™ 3-PLEX microfluidic chips (#402014, SynVivo Inc.) consisting of paired vascular (200 μm) and tissue (500 μm) channels separated by a 3 × 3 μm porous interface were used. Devices were coated with 200 μg/mL human fibronectin (#FC010, Millipore Sigma) for 1 h at 37 °C, rinsed with serum-free endothelial cell medium, and degassed by applying 7 psi nitrogen for 12 min to remove air bubbles. Primary human brain microvascular endothelial cells (HBMVECs, passage 1; #10HU-051, iXCells Biotechnologies) were suspended at 3 × 10^7^ cells/mL in complete endothelial cell growth medium (ECGM; #390598, R&D Systems). Cells were introduced into the vascular channel at 3 μL/min for 30 s, followed by static incubation for 4 h at 37 °C. A stepped flow regimen was then applied (0.02 → 0.05 μL/min over 8 h), transitioning to constant flow (0.05 μL/min) for 12 h. Primary astrocytes (#1800, ScienCell) and pericytes (#1200, ScienCell) were mixed at a 2:1 ratio at 1 × 10^7^ cells/mL in respective growth media and introduced into the tissue channel at 3 μL/min for 30 s. After 4 h of static incubation at 37 °C, a stepped flow regimen (0.05 → 0.1 μL/min over 8 h) was initiated, followed by constant perfusion (0.1 μL/min) for 32 h. Endothelial morphology, lumenization, and barrier integrity were assessed by inverted phase-contrast microscopy (Nikon Ti2 Eclipse) (Fig. 4*E*). rPOMC (3 ng/mL) or postrun sEVs (10^10^ particles/mL) were diluted in ECGM and introduced into the vascular channel at 0.1 μL/min for 4 h. Following exposure, effluents were collected from the tissue channel by perfusion with serum-free medium at 1 μL/min for 25 min. Samples were snap-frozen at −80 °C until analysis.

### Endothelial Passage and Melanin Assay.

30,000 human umbilical vein endothelial cells (HUVECs; #C0035C, Thermo Fisher Scientific) were seeded into the inserts of a 24-well Transwell plate (#07-200-154, Thermo Fisher Scientific) and cultured in HUVEC medium (phenol red-free Human Large Vessel Endothelial Cell Basal Medium, #M200PRF500, Thermo Fisher Scientific) supplemented with 10% Large Vessel Endothelial Supplement (#A1460801, Thermo Fisher Scientific). In parallel, 100,000 B16-F10 melanoma cells (#CRL-6475, ATCC) were seeded into the wells of the same plate and maintained in B16 medium (phenol red-free RPMI-1640, #11835030, Thermo Fisher Scientific) supplemented with 10% fetal bovine serum (FBS, #26140079, Thermo Fisher Scientific), 100 U/mL penicillin, and 100 µg/mL streptomycin (#15140122, Thermo Fisher Scientific). After confirming by microscopy that HUVECs had formed a confluent monolayer, inserts were transferred into the wells containing B16-F10 cells to initiate the coculture. Different concentrations of rPOMC or plasma-derived postrun sEVs were then added to the upper chamber. For the EV condition, the equivalent POMC concentration was measured by ELISA (see *ELISA* section). After 4 h, the concentration of POMC that passed into the lower chamber was quantified by ELISA for both rPOMC- and EV-treated groups. The cultures were then incubated for an additional 48 h to allow B16-F10 cells in the lower chamber to produce melanin in response to POMC passed through the endothelial layer. Following this incubation, melanin was extracted using 1 N NaOH containing 10% DMSO and incubated at 80 °C for 90 min (60). Absorbance at 490 nm was measured using the Varioskan Flash plate reader to assess melanin content. As a positive control, separate B16-F10 cultures were treated with α-melanocyte–stimulating hormone (α-MSH, #05-23-0751, Millipore Sigma) at different concentrations for 48 h under identical conditions. In all, untreated cells served as negative controls.

### pH-Dependent Incubation and Analysis of sEVs with Recombinant POMC.

The pH of plasma was adjusted by mixing at 1:1 volume ratio with pH-adjusted PBS to reach the target pH values of 7.40, 7.25, 7.10, 6.95, and 6.80. Samples were incubated at 37 °C for 50 min under 5% CO_2_ atmosphere to allow for stabilization of pH conditions. sEVs were then isolated following the standard protocol described above. To ensure consistency, the PBS used to resuspend the sEV pellet was preadjusted to match with a given pH condition. For specific experiments, rPOMC at various concentrations was added to B16-F10-derived sEVs and incubated under a determined pH condition. In other experiments, rPOMC (3.75 ng/mL) was added to sEVs isolated from SW480 and HDF cells in PBS buffer at pH 7.4 and incubated under similar conditions. All samples were subsequently analyzed via d-STORM as previously described.

### MD Simulations.

MD simulations were performed by using AMBER 18 (61). Since the experimental structure of full-length human POMC was not available, the 3D model was retrieved from the AlphaFold repository (62) (AF-P01189-F1) and used as 3D structural coordinates. The structure was protonated at two different pH values (7.0 and 7.4) using the H++ webserver (62) and optimized by running two replica MD simulations for 1 μs each. The ff14SB force field was used to parameterize the protein. The structures were solvated in a TIP3P water box and neutralized adding an appropriate number of Cl− ions. The generated systems were subjected to simulated three-step energy minimization involving first the hydrogens atoms, then the water molecules, and finally the protein side chains. Subsequently, a heating phase of 20 ps was carried out to gradually increase the temperature from 0 to 300 K by using the Langevin thermostat and applying positional restraints (5 kcal/mol) on the Cα atoms.

Two equilibration steps were performed using first the NVT ensemble for 50 ps, maintaining the positional restraints on Cα atoms, and then in NPT ensemble for 70 ps keeping the pressure around 1 atm by using the Berendsen barostat and gradually reducing the weight of the restraints. Two replicates of 1 μs each were performed in NPT ensemble without any restraints for both systems, protonated at pH 7.0 and 7.4. Electrostatic interactions were computed by the particle–mesh Ewald method and periodic boundary conditions were applied. For each simulation, the quality of the final structure was assessed by generating the corresponding Ramachandran plot by means of Procheck tool (63). The resulting refined structures were subjected to further 1 μs MD simulations, by using the same protocol described above. Resulting trajectories were then employed to perform the analyses by means of the Cpptraj module (64) of AmberTools18. Cluster analysis was performed based on the backbone atoms of the protein segments corresponding to ACTH, β-MSH, and γ-MSH, by using the average linkage hierarchical agglomerative method implemented in Cpptraj with an epsilon value of 3. The solvent accessible surface areas (SASA) analysis was carried out through the gmx sasa module of GROMACS v2021.3 (65) using the default parameter settings.

### Protein–Protein Docking.

Protein–protein docking was performed using the 3D structures of MC1-R (PDB ID 7F4D) (66), MC2-R (PDB ID 8GY7) (67), MC3-R (PDB ID 8IOC) (68), MC4-R (7F53) (69) and MC5-R (8INR) (68). The receptor structures were protonated at pH 7.0 and 7.4 by means of H++ webserver. Considering that MC-Rs are transmembrane proteins, each structure was embedded in a bilayer composed of 70% phosphatidylcholine and 30% cholesterol, by using CHARMM-GUI webserver (70), to account for the presence of the membrane. The docking simulation was performed by means of GRAMM software (71) and involved POMC conformations, obtained from cluster analysis, which appeared for at least 10% of the simulation time and using a grid step of 3 and a rotation angle of 30°. 1,000 poses were generated for each structure. The distance between Arg227, belonging to the binding motif HFRW of β-MSH, and the aspartate residue which is known to interact with this residue of MSHs (namely Asp117 in MC1-R, ASP107 in MC2-R, Asp121 in MC3-R, Asp126 in MC4-R and Asp119 in MC5-R) was evaluated for all the obtained binding modes in order to evaluate the ability of the full-length POMC to interact with the different MC-R isoforms through the segment corresponding to β-MSH.

### Statistical Methods.

Differences among time points in the study were incorporated into a simple repeated measures linear model which included participant as the subject effect, and time as a fixed within-subject effect fit using a generalized least squares model. The Akaike Information Criterion was used to identify the best covariance matrix structure and variance assumptions for log-transformed POMC (general correlation structure grouped by participant), size (first-order autoregressive structure, allowing different variances across time points), and concentration (compound symmetry correlation structure) (72) (Fig. 1). Pairwise comparisons are based on Bonferroni post hoc tests. POMC estimates were backtransformed. The experiments involving d-STORM and ELISA were done on a quasi-randomly chosen subset of runners at each time point, so comparisons were made using independent *t*tests (Fig. 1).

Prevalence of receptors on sEVs, and POMC associated with particular receptors on sEVs, was counted within the relevant population (Fig. 2). Comparisons between prerun and postrun were made using a generalized linear model with an overdispersed binomial distribution. Comparisons across pH levels for POMC association with sEVs were modeled in a one-way ANOVA with Bonferroni post hoc (Fig. 5) or a two-tailed *t* test comparing changes in two different pH conditions (Fig. 5). CI for receptor prevalence (Fig. 2), the prerun cell source relative to receptors (Fig. 3), and other proportion data (Fig. 3) were computed as Wilson score intervals (73). Comparisons between prerun and postrun POMC expression by cell source were modeled using a chi-squared test for multiple proportions (Fig. 3). Analyses were done using R v4.3.0 (74) and GraphPad Prism v10.0.0 for Windows, GraphPad Software, Boston, MA, www.graphpad.com.

## Supplementary Material

Appendix 01 (PDF)

## Data Availability

Study data are included in the article and/or *SI Appendix*.
