# Supplementary material for: Physical exercise increases binding of POMC to blood extracellular vesicles
Source: Proc Natl Acad Sci U S A. 2025 Dec 16;122(51):e2525044122. doi: 10.1073/pnas.2525044122 (PMC12745691; doi:10.1073/pnas.2525044122)
Supplement: Supplementary file 1 — Appendix 01 (PDF) [file pnas.2525044122.sapp.pdf]

PNAS

Supporting Information for

Physical Exercise Increases Binding of POMC to Blood Extracellular Vesicles

Mark F. Santos, Jacqueline Randa, Derek Tai, Giulio Vistoli, Nofar Avihen Schahaf, Serena Vittorio, Geily Fuentes, Rita Lauro, Sheila Mosallaei, Jana Karbanová, Alexandra M. K. Yokomizo, Denis Corbeil, Cheryl E. Hightower and Aurelio Lorico

Corresponding authors:

Aurelio Lorico
Email: alorico@touro.edu

Cheryl E. Hightower
Email: cheryl.vanier@imgen-research.com

This PDF file includes:

Supporting Methods

Figures S1 to S6

Table S1

Extended Methods

Subjects and IRB

Blood samples (5 mL) were drawn into K2 EDTA vacutainer tubes (#BD-367863, VWR) at three specific time points: prior to running, immediately post-run, and after 30 minutes of rest. Participants were required to abstain from running or engaging in strenuous physical activity for at least 24 hours prior to the trial and to avoid over-the-counter medications or dietary supplements for one week. Restrictions also included abstaining from alcohol for 72 hours, caffeine or nicotine for four hours, and food for two hours before the trial. Water consumption was allowed freely before and during the trial. On the trial day, participants were screened for drug use using a multi-drug urine test cup (#TDOA-7125, Prime Screen), and only those with negative results were included.

Immunoblotting

PVDF membranes were blocked in PBS with 1% bovine serum albumin (BSA; #001-000-162, Jackson ImmunoResearch) for one hour at RT and probed overnight at 4°C with primary antibodies (detailed in Supplemental Table S1). Afterward, secondary AlexaFluor^{488}-conjugated antibodies were added for 30 minutes at RT. Membranes were washed and visualized using the iBright FL1000 imaging system (Thermo Fisher Scientific).

Enzyme-Linked Immunosorbent Assay (ELISA)

Plasma samples and sEVs were added directly to the pre-coated wells of the ELISA plate. Standards and samples were incubated at 37°C for 2 hours to allow for antigen binding. The detection process employed a biotin-conjugated primary antibody followed by incubation with avidin-horseradish peroxidase (HRP). Substrate solution was added, and the enzyme-substrate reaction was allowed to proceed for 25 minutes before terminating with stop solution. All reagents were supplied in the kit. Optical density (O.D.) at 450 nm was measured using the Varioskan Flash plate reader (Thermo Fisher Scientific). A standard curve generated from known concentrations (included in the kit) was used to calculate sample concentrations.

Flow Cytometry

The buffy coat containing white blood cells was isolated from blood samples by centrifuging at 2,000 × *g* for 10 minutes at RT. White blood cells were fixed in 4% paraformaldehyde for 15 minutes and immunolabeled with primary antibodies targeting specific cell markers (detailed in Supplemental Table S1) for 1 hour on ice.

Extended Figures

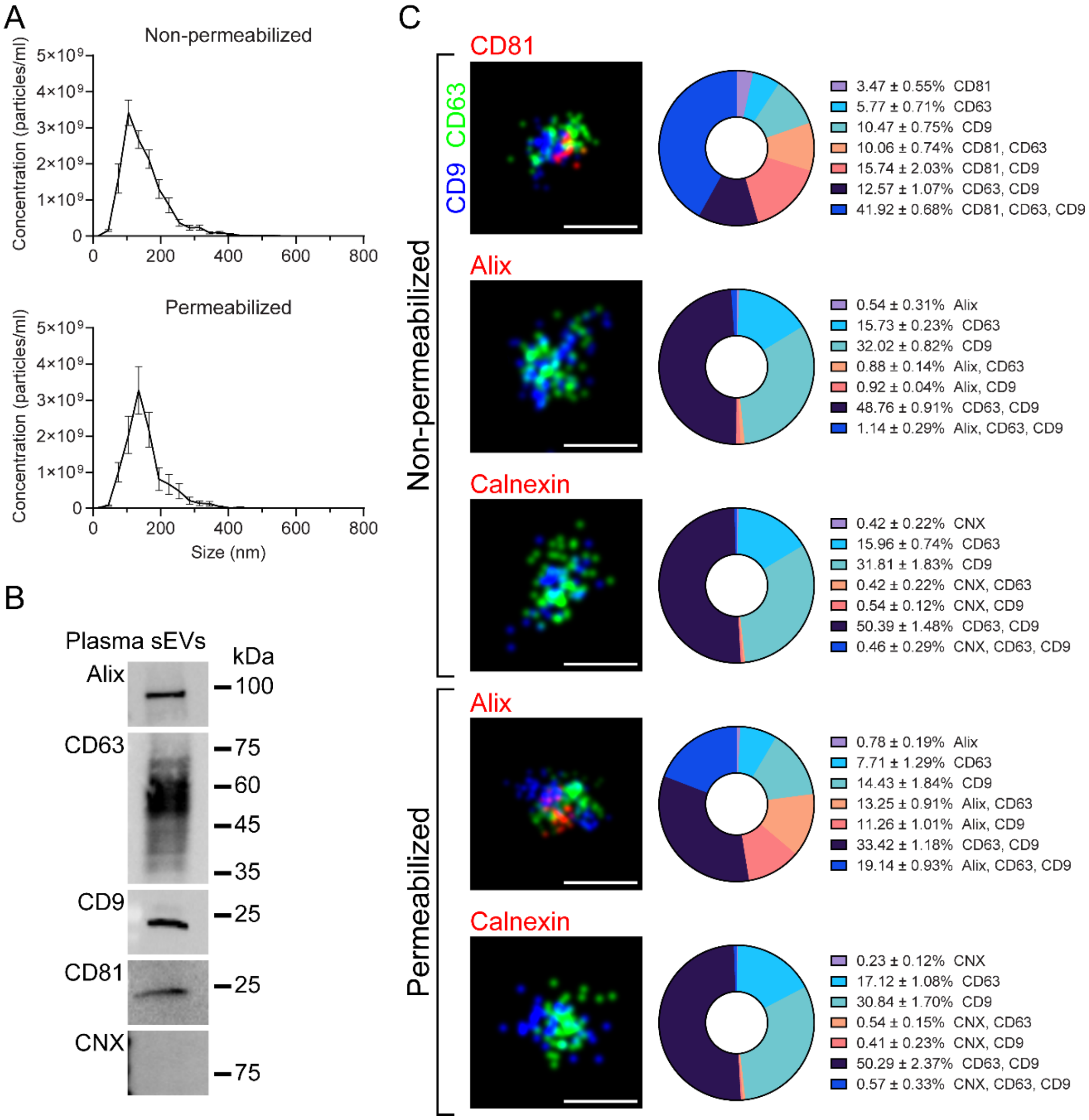

Extended Data Figure 1. Characterization of sEVs isolated from human peripheral blood of healthy volunteers. (A) EV size and concentration from pre-run samples (n = 6 independent participants) were analyzed using nanoparticle tracking analysis (NTA) in non-permeabilized and permeabilized samples. Mean ± S.E.M. are presented. (B) Immunoblot analysis of plasma-derived sEVs for Alix, CD63, CD9, CD81, and calnexin (CNX). The absence of CNX serves as a negative control, confirming the purity of the sEV preparations. (C) Non-permeabilized and permeabilized sEVs were immunolabeled for CD9 (blue), CD63 (green), and either CD81, Alix, or calnexin (red), followed by imaging

using d-STORM. Representative images are shown for each condition. The distribution of sEVs expressing triple, double, or single positivity for each marker is quantified and presented as mean ± S.E.M. (n = 3 independent experiments). Scale bars, 50 nm.

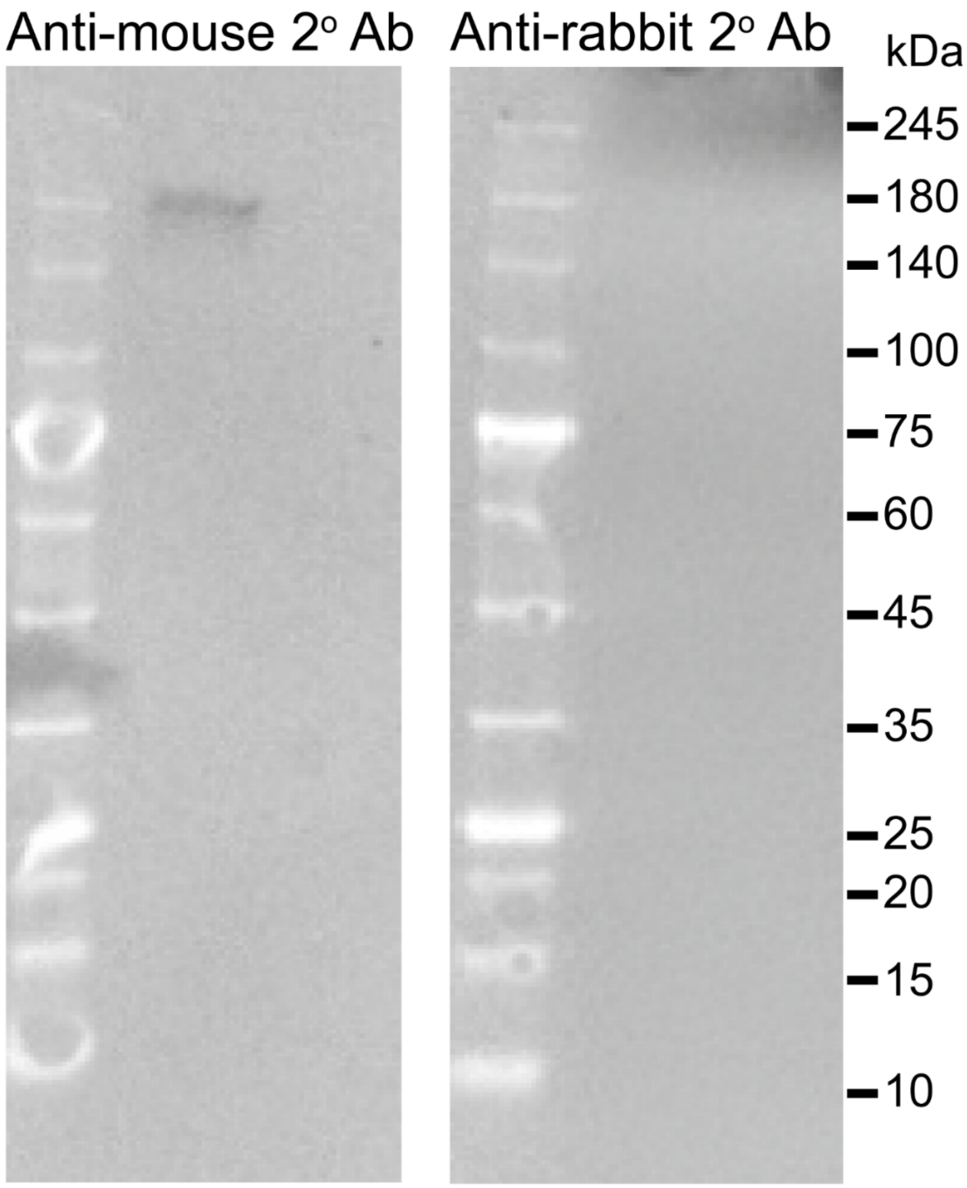

Extended Data Figure 2. Specificity of secondary antibodies against sEV samples. Immunoblot analysis of sEVs isolated from runners' plasma, probed using the secondary antibodies (anti-mouse or anti-rabbit) without primary antibodies. Note the lack of visible bands, confirming little to no background immune reactivity from the secondary antibodies used throughout the study.

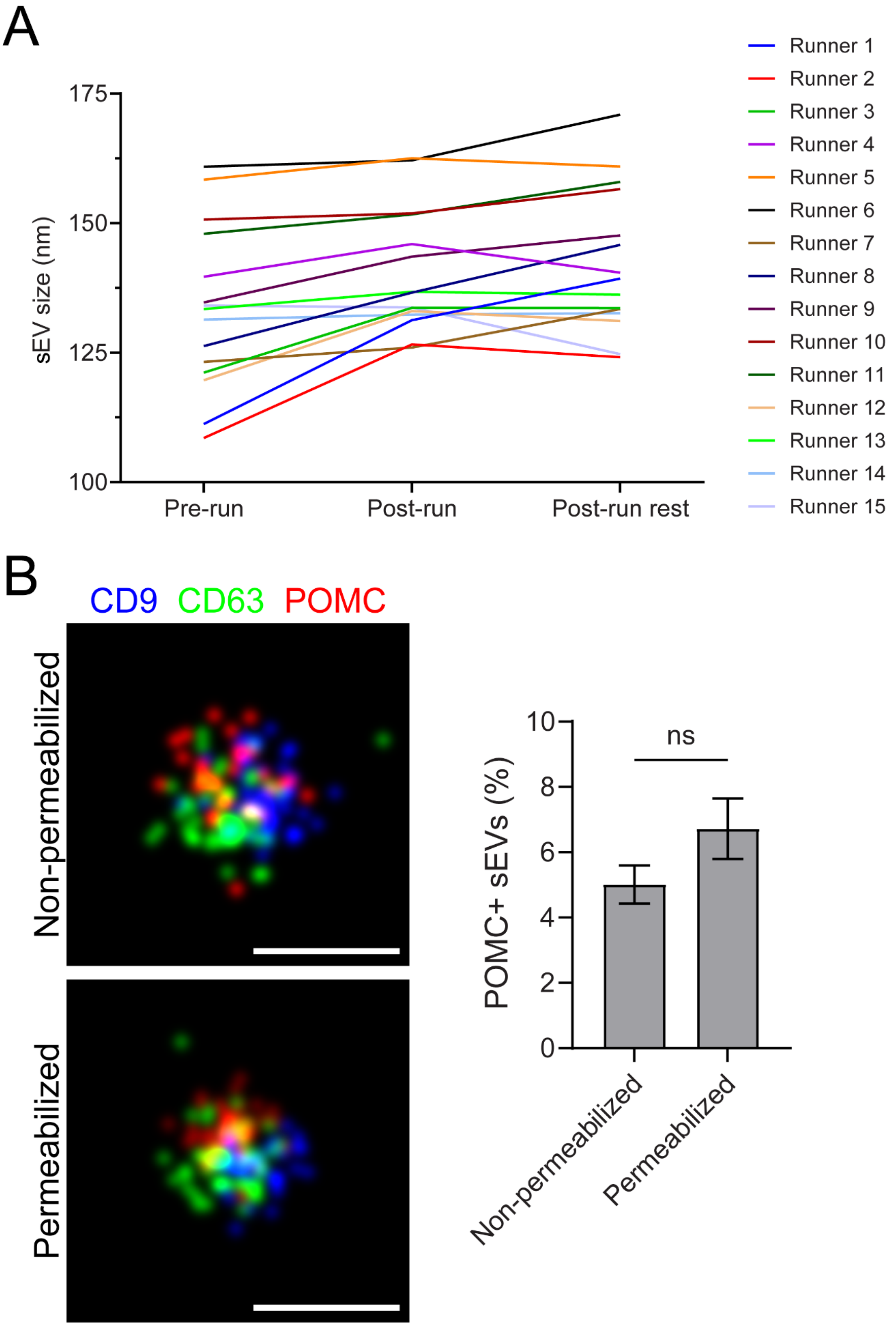
A
175
150
125
100
sEV size (nm)
Pre-run
Post-run
Post-run rest
Runner 1
Runner 2
Runner 3
Runner 4
Runner 5
Runner 6
Runner 7
Runner 8
Runner 9
Runner 10
Runner 11
Runner 12
Runner 13
Runner 14
Runner 15
B
CD9 CD63 POMC
Non-permeabilized
Permeabilized
10
8
6
4
2
0
ns
POMC+ sEVs (%)
Non-permeabilized
Permeabilized

Extended Data Figure 3. The number of sEVs in circulation did not change after physical exercise and POMCs bind to the surface of sEVs. (A) Size of sEVs isolated from the blood of runners before running, after a 50-minute run, and after a 30-minute rest, measured using NTA. Graph shows individual data for each runner (n = 15). (B) Enriched sEVs from plasma of healthy volunteers were immobilized on microfluidic slides, fixed, and either permeabilized or left non-permeabilized prior to immunolabeling for CD9, CD63, and POMC as indicated. Representative d-STORM images are shown for both conditions. Quantification of the percentage of $POMC^+$ sEVs (n = 3 runners, mean ± S.E.M.) revealed no statistically significant difference between permeabilized and non-permeabilized samples. Scale bars, 50 nm.

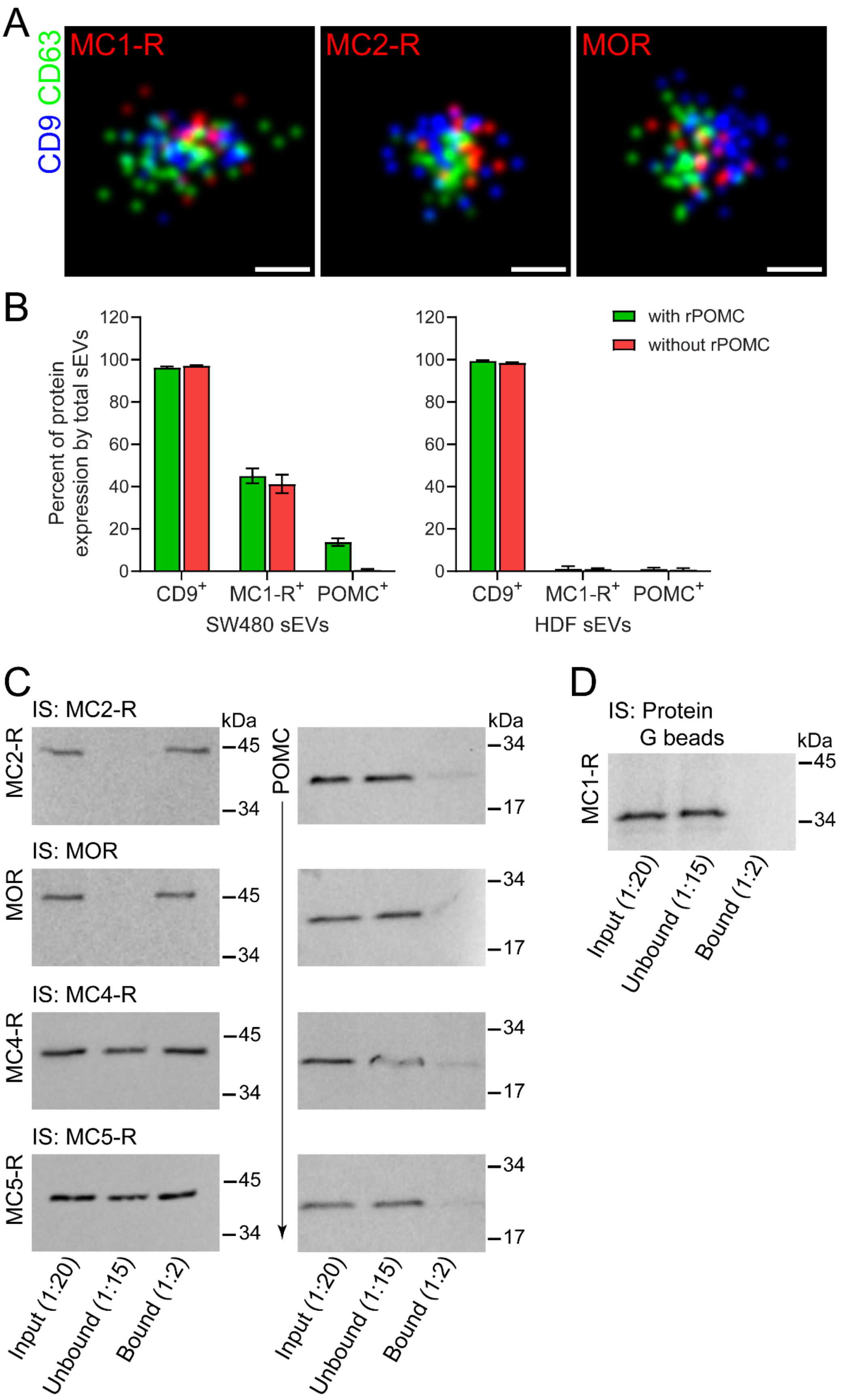
A
CD9 CD63
MC1-R
MC2-R
MOR
B
Percent of protein expression by total sEVs
120
100
80
60
40
20
0
with rPOMC
without rPOMC
CD9+
MC1-R+
POMC+
SW480 sEVs
HDF sEVs
C
IS: MC2-R
MC2-R
IS: MOR
MOR
IS: MC4-R
MC4-R
IS: MC5-R
MC5-R
POMC
kDa
45
34
17
Input (1:20)
Unbound (1:15)
Bound (1:2)
D
IS: Protein G beads
MC1-R
kDa
45
34
Input (1:20)
Unbound (1:15)
Bound (1:2)

Extended Data Figure 4. POMC binds preferentially to MC1-R. (A) Enriched sEVs from plasma were surface immunolabeled for CD9 (blue), CD63 (green), and either MC1-R, MC2-R, or MOR (red) and imaged using d-STORM. Scale bars, 50 nm. (B) sEVs derived from SW480 and HDF cells were incubated with or without recombinant POMC (rPOMC) for 50 minutes and analyzed by d-STORM. The percentage (mean ± S.E.M.) of sEVs positive for CD9, MC1-R, and POMC is shown from three independent experiments (n = 3). HDF-derived sEVs, which do not express MC1-R, showed no detectable POMC binding. In contrast, SW480-derived sEVs, which express MC1-R, demonstrated modest binding of POMC (<15%). (C) sEVs from post-run plasma were solubilized and subjected to immunoisolation (IS) for MC2-R, MC4-R, MC5-R, or MOR. Immunoblotting was performed on input, unbound, and bound fractions to assess the presence of POMC and the respective receptor. Trace amounts of POMC were detected in the bound fractions, consistent with the smaller increases observed by d-STORM (Fig. 2C). (D) Negative control for immunoisolation. sEVs from post-run plasma were incubated with Protein G beads without primary antibody. Immunoblotting for MC1-R showed no detectable signal, confirming that Protein G beads alone do not pull down receptors.

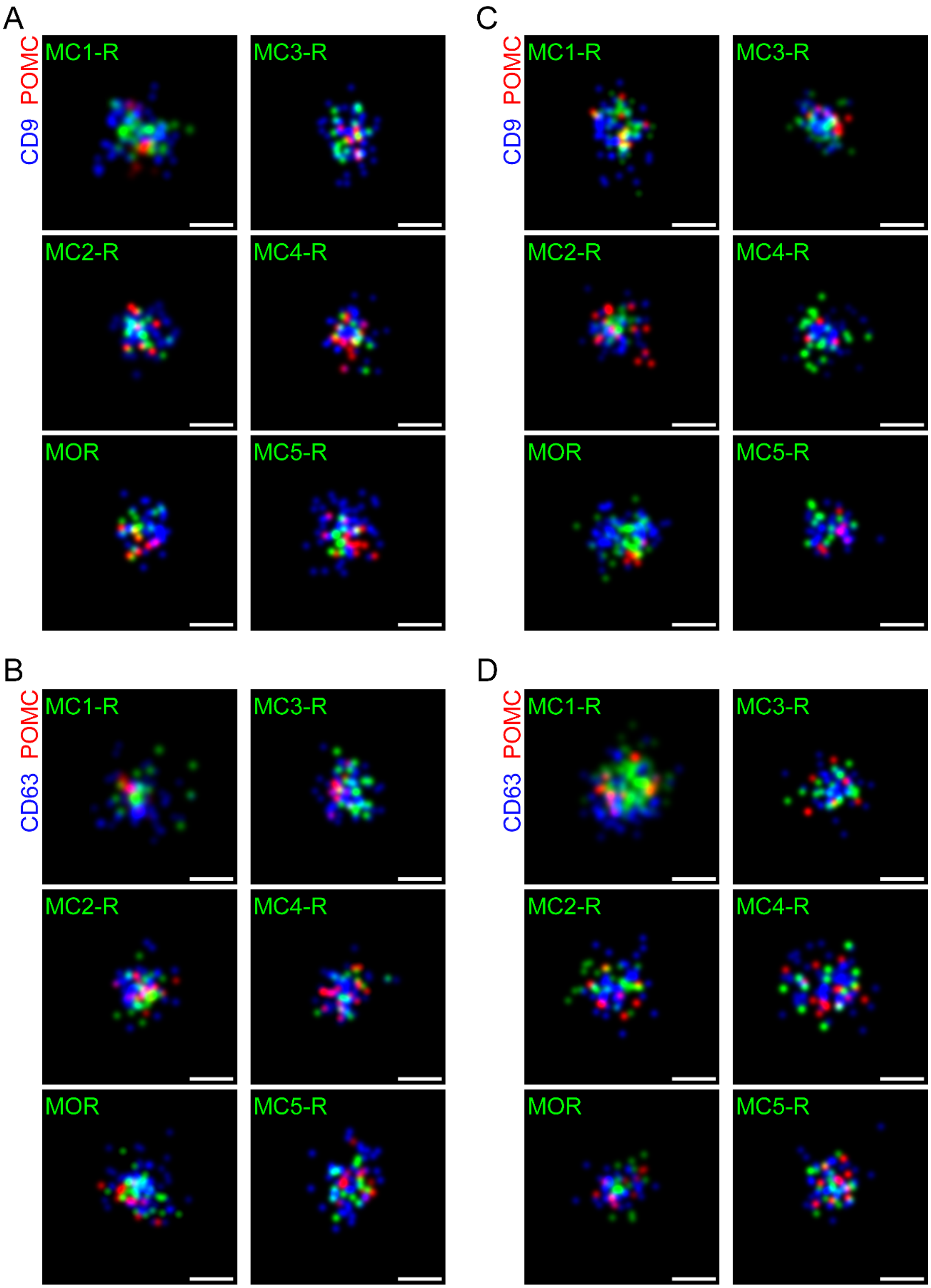

Extended Data Figure 5. Plasma-derived sEVs express melanocortin receptors on their surface. (A–D) Enriched sEVs from plasma were immunolabeled for POMC (red),

CD9 (blue, A and C) or CD63 (blue, B and D), and melanocortin receptor markers (MC1-R, MC2-R, MOR, MC3-R, MC4-R, or MC5-R; green) and analyzed using d-STORM. sEVs were isolated before (A, B) and after exercise (C, D). Scale bars, 50 nm.

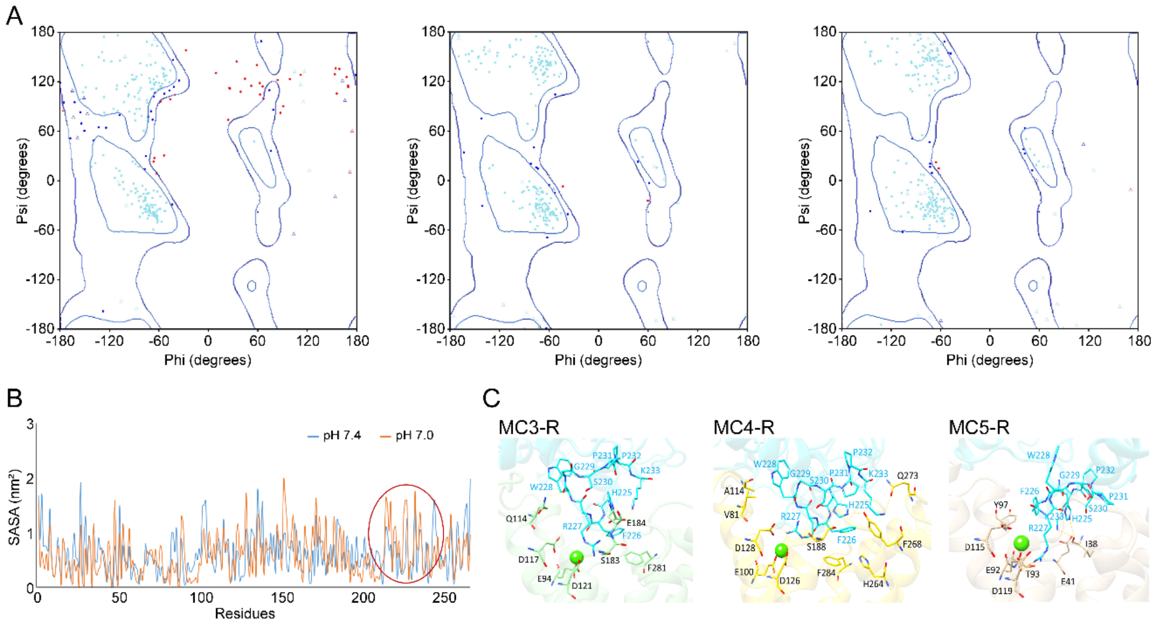

Extended Data Figure 6. The binding affinity of POMC is dependent on pH level. (A) Ramachandran plots for the starting AlphaFold model (left) and for the frame at the end of the first microsecond of the molecular dynamics (MD) runs at pH 7.4 (middle) and 7 (right). Cyan, blue, and red (dots/triangles) represent torsion angles of favored, allowed, and disallowed regions, respectively. Dots represent residues other than glycine and triangles represent glycine. In the AlphaFold model, 71.70% residues are located in the favored regions, 14.72% in allowed regions, and 13.59% in disallowed regions. After 1 µs MD simulation, the structure protonated at pH 7.4 contains 92.21% residues within favored regions, 6.93% in allowed regions, and 0.87% in disallowed regions. After 1 µs MD simulation, the structure protonated at pH 7.0 contains 91.21% residues in favored regions, while 7.53% and 1.26% residues are within allowed and disallowed regions, respectively. (B) Per-residue solvent accessible surface for the POMC residues as computed by averaging the values obtained from the two molecular dynamics replicas at pH 7.4 and 7.0. The red circle highlights the segment corresponding to β-MSH. (C) Putative complexes of the protonated POMC structure with MC3-R (left), MC4-R (middle) and MC5-R (right). Notice that in all three complexes, the β-MSH segment is suitably accommodated within the receptor binding site, and Arg227 of POMC is involved in the key salt bridge interacting with Asp121 in MC3-R, Asp126 in MC4-R, and Asp119 in MC5-R.

Supplemental Table S1. List of antibodies.

Target	Clone (host species)	Catalog number	Manufacturer	Application (dilution)
ACTH	CLIP/1407 (m)	NBP2-47658	Novus Biologicals	WB (1:500)
ACTH-Alexa Fluor 647	CLIP/1418 (m)	NB030072	Fisher Scientific	IF (1:100)
ACTHR	Polyclonal (r)	bs-11408R	Bioss	WB (1:500) IS (1:250)
ACTHR-Alexa Fluor 555	Polyclonal (r)	bs-11408R-A555	Bioss	IF (1:100)
ACTHR-Alexa Fluor 647	Polyclonal (r)	bs-11408R-A647	Bioss	IF (1:100)
Alix	3A9 (m)	2171	Cell Signaling Technology	WB (1:500)
Alix-Alexa Fluor 647	3A9 (m)	NB100-65678AF647	Novus Biologicals	IF (1:100)
β-Endorphin	20H4L2 (r)	PI701295	Fisher Scientific	WB (1:500)
β-Endorphin-Alexa Fluor 647	B31.15 (m)	NB500-414AF647	Novus Biologicals	IF (1:100)
Calnexin	AF18 (m)	MA3027	Fisher Scientific	WB (1:500)
Calnexin-Alexa Fluor 647	AF18 (m)	PIMA3027AY	Fisher Scientific	IF (1:100)
CD14-Alexa Fluor 488	63D3 (m)	367130	BioLegend	FC (1:100)
CD3-Alexa Fluor 488	OKT3 (m)	50-112-9566	Fisher Scientific	FC (1:100)
CD63	Ts63 (m)	10-628-D	Fisher Scientific	WB (1:500)
CD66b-FITC	G10F5 (m)	555724	BD Biosciences	FC (1:100)
CD81	1.3.3.22 (m)	PIMA513548	Fisher Scientific	WB (1:500)
CD9	P1/33/2 (m)	sc-20048	SantaCruz Biotechnology	WB (1:500)
MC1R	6D1S5 (r)	NBP3-15409	Novus Biologicals	WB (1:500) IS (1:250)
MC1R-Alexa Fluor 488	Polyclonal (r)	bs-23517R-A488	Bioss	IF (1:100)
MC1R-Alexa Fluor 555	Polyclonal (r)	bs-23517R-A555	Bioss	IF (1:100)

MC1R-Alexa Fluor 647	Polyclonal (r)	bs-23517R-A647	Bioss	IF (1:100)
MC3R	Polyclonal (r)	NBP3-10027	Novus Biologicals	WB (1:500)<br>IS (1:250)
MC3R-Alexa Fluor 488	345404 (r)	FAB3737G	RnD Systems	IF (1:100)
MC3R-Alexa Fluor 555	Polyclonal (r)	bs-7558R-A555	Bioss	IF (1:100)
MC4R	Polyclonal (r)	NBP2-41103	Novus Biologicals	WB (1:500)<br>IS (1:250)
MC4R-Alexa Fluor 555	Polyclonal (r)	bs-11417R-A555	Bioss	IF (1:100)
MC5R	JE61-09 (r)	PIMA544865	Fisher Scientific	WB (1:500)<br>IS (1:250)
MC5R-Alexa Fluor 555	Polyclonal (r)	bs-23517R-A555	Bioss	IF (1:100)
MC5R-Alexa Fluor 647	821012 (m)	FAB8205R	RnD Systems	IF (1:100)
MSH gamma	Polyclonal (r)	bs-3918R	Bioss	WB (1:500)
μ Opioid Receptor	Polyclonal (r)	NBP1-31180	Novus Biologicals	WB (1:500)<br>IS (1:250)
μ Opioid Receptor-Alexa Fluor 555	Polyclonal (r)	bs-3724R-A555	Bioss	IF (1:100)
μ Opioid Receptor-Alexa Fluor 647	Polyclonal (r)	bs-3724R-A647	Bioss	IF (1:100)
POMC	EPR17571 (r)	ab210605	Abcam	WB (1:500)
POMC[a]	6D2B5 (m)	PIMA538604	Fisher Scientific	IS (1:500)
POMC-Alexa Fluor 647[b]	OTI2B2 (m)	NBP2-73525AF647	Novus Biologicals	IF (1:100)
Anti-mouse IgG-Alexa Fluor 488	Polyclonal (g)	A11017	Fisher Scientific	WB (1:2000)
Anti-rabbit IgG-Alexa Fluor 488	Polyclonal (g)	A11070	Fisher Scientific	WB (1:2000)

m, mouse; r, rabbit

WB, Western blot; IF, immunofluorescence; IS, immunoisolation; FC, flow cytometry

[a]Generated against recombinant fragment of human POMC (aa: 1-150) expressed in E. coli. Specific binding epitope is not specified.

[b]*Generated against recombinant full-length human POMC (NP_001030333) produced in HEK293T cells. Specific binding epitope is not specified. Cross-reactivity against POMC-derived hormones was not tested by manufacturer.*
